# Metal-Halide Perovskite Submicrometer-Thick Films for Ultra-Stable Self-Powered Direct X-Ray Detectors

**DOI:** 10.1007/s40820-024-01393-6

**Published:** 2024-04-26

**Authors:** Marco Girolami, Fabio Matteocci, Sara Pettinato, Valerio Serpente, Eleonora Bolli, Barbara Paci, Amanda Generosi, Stefano Salvatori, Aldo Di Carlo, Daniele M. Trucchi

**Affiliations:** 1 CNR-ISM, Consiglio Nazionale delle Ricerche, Istituto di Struttura della Materia, Sede Secondaria di Montelibretti, DiaTHEMA Lab, Strada Provinciale 35D, 9, 00010 Montelibretti, Rome, Italy; 2 CHOSE – Centre for Hybrid and Organic Solar Energy, Department of Electronic Engineering, University of Rome ‘‘Tor Vergata’’, Via del Politecnico 1, 00133 Rome, Italy; 3 Faculty of Engineering, Università degli Studi Niccolò Cusano, Via don Carlo Gnocchi 3, 00166 Rome, Italy; 4 SpecXLab, CNR-ISM, Consiglio Nazionale Delle Ricerche, Istituto di Struttura Della Materia, Area della Ricerca di Tor Vergata, Via del Fosso del Cavaliere 100, 00133 Rome, Italy

**Keywords:** Metal-halide perovskite thin films, Direct X-ray detectors, Self-powered devices, Operational stability, Medical linear accelerator

## Abstract

Metal-halide perovskites are revolutionizing the world of X-ray detectors, due to the development of sensitive, fast, and cost-effective devices. Self-powered operation, ensuring portability and low power consumption, has also been recently demonstrated in both bulk materials and thin films. However, the signal stability and repeatability under continuous X-ray exposure has only been tested up to a few hours, often reporting degradation of the detection performance. Here it is shown that self-powered direct X-ray detectors, fabricated starting from a FAPbBr_3_ submicrometer-thick film deposition onto a mesoporous TiO_2_ scaffold, can withstand a 26-day uninterrupted X-ray exposure with negligible signal loss, demonstrating ultra-high operational stability and excellent repeatability. No structural modification is observed after irradiation with a total ionizing dose of almost 200 Gy, revealing an unexpectedly high radiation hardness for a metal-halide perovskite thin film. In addition, trap-assisted photoconductive gain enabled the device to achieve a record bulk sensitivity of 7.28 C Gy^−1^ cm^−3^ at 0 V, an unprecedented value in the field of thin-film-based photoconductors and photodiodes for “hard” X-rays. Finally, prototypal validation under the X-ray beam produced by a medical linear accelerator for cancer treatment is also introduced.

**Supplementary Information:**

The online version contains supplementary material available at 10.1007/s40820-024-01393-6.

## Highlights

- Self-powered direct X-ray detectors, based on FAPbBr_3_ 255-nm-thick films deposited onto mesoporous TiO_2_ scaffolds, can withstand a 26-day uninterrupted X-ray exposure with negligible signal loss, demonstrating ultra-high operational stability.
- Bulk specific sensitivity is evaluated to be 7.28 C Gy^−1^ cm^−3^ at 0 V, an unprecedented value in the field of thin-film-based photoconductors and photodiodes for “hard” X-rays.
- Sensitivity of submicrometer-thick perovskite films to the X-rays produced by a medical linear accelerator used for cancer treatment is here demonstrated for the first time.

**Supplementary Information:**

The online version contains supplementary material available at 10.1007/s40820-024-01393-6.

## Graphical abstract

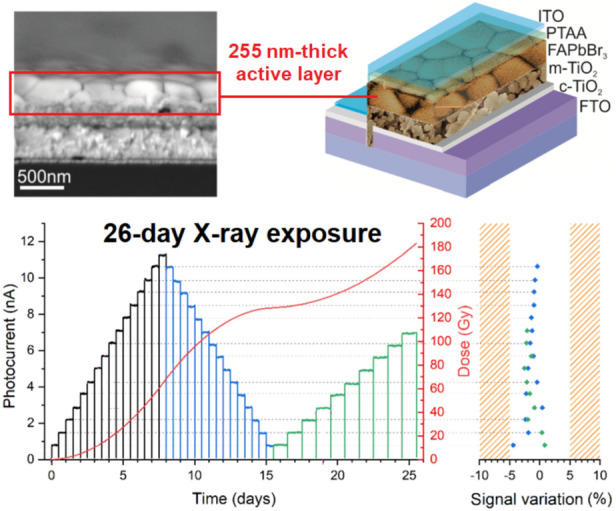

## Introduction

In recent years, metal-halide perovskites (MHPs) have emerged as an extremely promising candidate [1, 2] for the development of high-performance X-ray detectors, due to their unique combination of appealing properties (*e.g*., strong X-ray absorption, large charge carrier mobility–lifetime product, high resistivity, fast response, low production cost, and even the possibility of being deposited as thin films on flexible substrates). MHPs have been exploited both for direct detectors, where they directly convert X-ray photons into electric charge [3, 4], and for indirect detectors, where they are used as scintillators [5–7]. In both these cases, MHPs have demonstrated to potentially introduce a disruptive innovation in the world of X-ray detectors, not only from a merely performance-based point of view, but also concerning market-oriented aspects. For instance, perovskite direct detectors have been shown to outperform traditional Si or CZT-based devices in terms of sensitivity to X-rays [8, 9]. Additionally, X-ray imaging systems that utilize MHP-based scintillators provide equivalent spatial resolution to rare-earth- or heavy-metal-based systems, while drastically reducing production costs [10, 11].

Despite these encouraging results, MHP-based X-ray detectors are still relatively far from commercialization, mainly due to their limited stability over time. In most cases, this is caused by the use of volatile cations, such as methylammonium (MA), which if on the one hand results in outstanding surface sensitivity and extremely low limit of detection [12–14], on the other leads to ionic drift and lattice instability [15, 16]. As a first strategy to improve the device stability, self-powered operation (*i.e.,* without the application of an external bias) can be pursued, aimed at mitigating ion migration effects [17, 18]. Furthermore, self-powering is an essential milestone on the technology roadmap for thin-film-based flexible sensors [19]. It is indeed highly desirable for portable and wearable electronic devices, as well as for specific applications in harsh environments like nuclear power or space exploration, where energy availability is restricted. Another effective solution, aimed at reducing lattice instability, is to substitute some or all the MA cations with cesium (Cs), formamidinium (FA), or guanidinium (GA) [20–22], often adopting mixed-cation alloys, such as CsFA or CsFAGA [23], to enhance the device performance. The two strategies can also be pursued jointly to obtain X-ray detectors with improved stability over time, as recently demonstrated with MHP single crystals [24, 25]. However, the long-term reliability of perovskite-based X-ray detectors has mostly been assessed in terms of storage stability, *i.e.,* by periodically evaluating the device performance after a long-term storage period typically spanning several months [23–27], but the evaluation of operational stability, which focuses on confirming the presence of consistent and reproducible photocurrent signals under prolonged uninterrupted exposure to X-rays, has not received comparable attention. Up to now, the operational stability under continuous irradiation of X-ray detectors based on MHP bulk materials or thin films has only been tested up to a few hours, often reporting degradation of the detection performance [22, 23, 26, 28–32]. Only hybrid detectors based on perovskite-filled membranes (PFM) have been exposed to X-rays uninterruptedly for about 3.5 days, albeit exhibiting signal fluctuations up to 5% [33].

Here we show that, by combining the strategy of self-powering with the use of stable cations, MHP-based detectors can withstand a 26-day uninterrupted X-ray exposure with negligible signal loss, demonstrating ultra-high operational stability and excellent repeatability. The prototypal device introduced in this work has the structure of semi-transparent perovskite solar cells (ST-PSCs). Indeed, a solar cell, if proven both sensitive to X-rays and stable under irradiation for a long time, could in principle meet simultaneously the requirements of a self-powered X-ray detector with high operational stability. Very recently, MHP solar cells based on MAPbBr_3_ (methylammonium lead bromide) active layers deposited onto mesoporous TiO_2_ (m-TiO_2_) scaffolds have been reported to significantly improve the stability over time [34]. In this work, aimed at optimizing the X-ray detection performance, we replaced MA cations with FA cations, which are known to be more stable under X-rays, and tailored to FAPbBr_3_ (formamidinium lead bromide) the deposition technology of submicrometer-thick films on m-TiO_2_ scaffolds. It is shown that self-powering, ensuring an extremely low noise with no drift, in synergy with the superior chemical and structural stability of the FAPbBr_3_/m-TiO_2_ stack, are the key factors for the ultra-stable performance of the developed X-ray detector, as well as for its unexpectedly high (for a perovskite thin film) radiation hardness. We also demonstrate that trap-assisted photoconductive gain is the key to obtain a highly sensitive detector; a bulk sensitivity up to 7.28 C Gy^−1^ cm^−3^ has indeed been obtained, which is the highest value ever reported on thin-film-based photoconductors and photodiodes for “hard” X-rays. Finally, we show that the device can reliably operate in relevant environment, as assessed with a high-energy X-ray beam produced by a medical LINAC (linear accelerator) commonly used for radiation therapy to treat cancer, further demonstrating the high potentiality of submicrometer-thick FAPbBr_3_ films for X-ray detection.

## Experimental Section

### Device Fabrication

Fluorine-doped tin oxide (FTO) glass substrates (TEC 7, Pilkington) were etched by using a nanosecond-pulsed UV laser in order to obtain electrically insulated areas. The resulting substrates were cleaned in ultrasonic bath using sequentially acetone, ethanol and 2-propanol (5 min each). Then, a 30-nm-thick compact TiO_2_ (c-TiO_2_) layer was deposited by spray-pyrolysis at 460 °C. The c-TiO_2_ films were then used as substrates for the spin coating of 245 nm-thick mesoporous TiO_2_ (m-TiO_2_) films using a diluted (1:5 w/w in ethanol) screen-printing paste (30NR-D, Great Cell Solar Materials), followed by sintering at 480 °C for 30 min. Then, 255 nm-thick FAPbBr_3_ perovskite films were deposited by spin coating at 4000 rpm for 20 s using a dimethyl-sulfoxide (DMSO) solution (1.4 M) obtained by mixing FABr (Great Cell Solar Materials) and PbBr_2_ (TCI chemicals) precursors. Anhydrous ethyl acetate (Sigma Aldrich) was dropped as anti-solvent 10 s before the end of the spin coating process. After spin coating, the samples were kept on a hot plate at 80 °C for 10 min. Then, poly[bis(4-phenyl)(2,4,6-trimethylphenyl)amine] (PTAA) solution with concentration 10 mg mL^−1^ in toluene (10KDa, Solaris Chem), doped with 4-tert-butylpyridine (10 µL mL^−1^) and a lithium bis(trifluoromethanesulfonyl)imide (LiTFSI) solution (5 µL mL^−1^ of 180 mg mL^−1^) in acetonitrile (ACN), was spin-coated at 4000 rpm for 20 s. Finally, transparent indium tin oxide (ITO) electrodes were deposited by radio-frequency (RF) sputtering at low power density (0.39 W cm^−2^) and at a 1.1 × 10^–3^ mbar chamber pressure.

The electron-only device (see Sect. 3.3) used for SCLC measurements, TRPL analysis, and the evaluation of the mobility–lifetime product was fabricated by following the same procedure described above, excluding the steps followed for the PTAA hole transport layer. The same applies to the fabrication procedure of the device without the m-TiO_2_ scaffold (see Sect. 3.5), but excluding in this case the steps followed for the m-TiO_2_ layer deposition and sintering.

### Assessment of the Optical, Structural, and Morphological Properties

Planar and cross-sectional SEM images were acquired by using a field emission gun scanning electron microscope (FEG-SEM) system (TESCAN Analytics VEGA). Absorption coefficient was calculated from the absorbance spectra measured by using a UV–Vis spectrophotometer (Shimadzu UV-2550) equipped with an integrating sphere. Elemental energy-dispersive X-ray (EDX) maps were acquired by using the Xplore detector by Oxford Instruments (electron beam energy 10 keV). A PANalytical Empyrean diffractometer was used to perform X-ray diffraction (XRD) measurements in reflection mode. The *K*_α_ fluorescence lines (*K*_*α1*_ = 1.54060 Å, *K*_*α2*_ = 1.54443 Å) of a Cu anode were selected as impinging radiation and detected by a solid-state hybrid Pix’cel 3D detector working in 1D linear mode. High-angle XRD measurements were collected in the 10° < 2*θ* < 70° angular range, and Bragg Brentano configuration was adopted focusing the impinging beam with fixed divergent slits (1/4°–1/2°). Atomic force microscopy (AFM) measurements were performed by using an in-house-developed system mounting a 30 × 30 μm^2^ scanner. Non-contact mode acquisitions were collected upon several portions of each film by means of Al-coated standard tapping AFM probes (Nanosensors). Steady-state (PL) and time-resolved (TRPL) photoluminescence signals were acquired by using a multi-platform characterization system (Arkeo, Cicci Research srl) equipped with a UV laser (wavelength 375 nm) pump. Acquisition time for PL signals monitoring was set to 1 s.

### Characterization of the Device as a Solar Cell

Transmittance spectra were acquired by using a UV–Vis spectrophotometer (Shimadzu UV-2550) equipped with an integrating sphere. Sheet resistance of the sputtered ITO electrodes was measured by using a source-meter (Keithley 2620) with the four-point probe technique. *J-V* characterizations were performed by using a Class-A sun simulator (ABET 2000) equipped with an AM1.5G filter (ABET). The calibration of the sun simulator was made by using a Si-based reference cell (RR-226-O, RERA Solutions) in order to obtain 1 Sun illumination conditions. Current density at different bias voltages was measured by using an Arkeo platform (Cicci Research srl) able to measure the device under forward and reverse scan directions, with voltage steps of 50 mV and a scan rate of 300 mV s^−^^1^. The device was then polarized at maximum power point (MPP) for the measurement of the steady-state power conversion efficiency (*PCE*) with an acquisition time of 60 s.

### Experimental Setup for UV–Vis−NIR Spectral Photoconductivity

Light came out from the output slit of a dual grating monochromator (Newport Cornerstone 260) coupled to a Hg-Xe lamp, chopped at a frequency of 14 Hz by a SR540 mechanical chopper, and focused on the active area of the device. The photogenerated current signals at the different wavelengths were converted by a transimpedance amplifier (Princeton Applied Research 181) into a voltage signal measured by a lock-in amplifier (EG&G 5209). Power was measured by means of a calibrated Si-based photodiode (Newport 818-UV) and a power meter (Newport 843-R).

### Assessment of the Photoelectronic Properties in the UV–Vis−NIR Range

The responsivity *R*(*λ)* was calculated as a function of the impinging wavelength *λ* as *R*(*λ)* = *I*_*ph*_(*λ)/P*(*λ)*, where *I*_*ph*_ is the amplitude of the measured photocurrent and *P* is the power of the monochromatic radiation focused on the active area of the device. The external quantum efficiency *EQE* (*λ*) was derived from the responsivity *R*(*λ*) by applying the following equation: *EQE* (*λ*) = *R*(*λ*)*⋅* (*hc/λe*), where* h* is the Planck’s constant,* c* is the speed of light in vacuum, *e* is the electron charge.

The electron mobility–lifetime product (*μ*_*e*_*τ*_*e*_) was extracted by fitting the *I*_*ph*_ vs.*V*_B_ curve according to Hecht's equation [35]:

1 $${I}_{ph}\propto \frac{{\mu }_{e}{\tau }_{e}{V}_{B}}{{d}^{2}}\left[1-{\text{exp}}\left(-\frac{{d}^{2}}{{{\mu }_{e}{\tau }_{e}V}_{B}}\right)\right]$$

where *d* = 255 nm is the thickness of the FAPbBr_3_ active layer of the electron-only device and *V*_B_ is the applied bias voltage.

The specific detectivity D* was calculated as:

2 $$\it {D}^{*}\left(\lambda \right)=\frac{\sqrt{AB}}{{{NEP}}}=\frac{R(\lambda )\sqrt{A}}{{i}_{{{noise}}}}$$

where *A* = 0.4 cm^2^ is the active area of the device, *B* is the detection bandwidth, *NEP* = *i*_*noise*_/*R*(*λ)* is the noise equivalent power, calculated from the device responsivity *R*(*λ)* and the total noise current *i*_*noise*_ of the detection system. The total noise current *i*_*noise*_ was derived in the 0.1–50 Hz frequency range by applying a fast Fourier transform (FFT) [36] to the dark current *I*_*d*_ measured as a function of time (and at *V*_B_ = 0 V) by a Keithley 487 electrometer (integration time 10 ms). The same electrometer was used also to measure dark current as a function of the applied bias voltage. The dark current density was obtained as *J*_*d*_ = *I*_*d*_*/A.*

Capacitance–voltage measurements were performed in the  ± 1.5 V range by means of a Hewlett Packard 4192A LF impedance analyzer, with a probing AC signal of 30 mV at a frequency of 3 kHz.

### Experimental Setup for Characterization under Continuous keV-Range X-Rays

X-ray photoresponse was investigated under continuous X-rays by means of a Coolidge tube equipped with a Cu target. No filter was interposed between the X-ray source and the device. In this way, in addition to the 8.05 keV (*K*_*α*_) and 8.91 keV (*K*_*β*_) lines emitted by the Cu target, also the *Bremsstrahlung* radiation impinged the detector. The device was placed at a distance of 10 cm from the output slit of a collimator (1 mm in diameter), resulting in a circular spot size on the active area of about 5 mm^2^ (as measured with a fluorescent screen), and then connected to a Keithley 487 electrometer, simultaneously used as a voltage supply (bias voltage was set to 0 V) and a current meter. The tube acceleration voltage was set to 40 kV, thus producing X-ray photons with energy up to 40 keV. The dose rate (*D*) of the emitted X-rays was adjusted by varying the tube current in the range 0.15–40 mA, resulting in an X-ray dose rate varying in the range 644 nGy s^−1^–189.3 µGy s^−1^, as measured in a reference ionization chamber (Farmer mod. NE 2536/3C) by a commercial dosimeter (Farmer mod. NE 2670). Aimed at eliminating any possible contribution of temperature or humidity variations to the photocurrent signal stability, all X-ray measurements have been performed inside a shielded X-ray room specifically designed for low-noise experiments. Experiments were all remote-controlled, and performed under controlled conditions of relative humidity and temperature (22 °C and 40%, respectively, which are the average recommended values for hospital radiology and radiotherapy rooms). Specifically, the temperature difference between the sample holder and the inner wall of the X-ray room was constantly monitored by two thermocouples, and the related information was fed back to the control unit of the air conditioning system.

### Assessment of Detection Properties under Continuous keV-Range X-Rays

*SNR* was calculated as the ratio between the X-ray average photocurrent measured at each dose rate value and the standard deviation of the dark current, as reported in the following equation:

3 $${\it{SNR}}=\frac{\frac{1}{n}\sum_{i}^{n}{I}_{{ph}_{i}}}{\sqrt{\frac{1}{n}\sum_{i} ^{n}{({{I}_{d}}_{i}-{\overline{I} }_{d})}^{2}}}$$

where* n*, appearing in the numerator (denominator), is the number of acquisitions during the “X-ray on” (“X-ray off”) period, and ${\overline{I} }_{d}$ is the average dark current.

The surface specific sensitivity, defined as *S*_*s*_ = *I*_*ph*_/(*A⋅ D*), was evaluated from the slope of the linear fitting curve of photocurrent vs. dose rate data. The bulk specific sensitivity (*i.e*., the specific sensitivity normalized to the active volume) was calculated as *S*_*v*_ = *S*_*s*_*/d*.

The X-ray photocurrent pulse rise time *t*_*r*_ was defined as the time required for the pulse to rise from 10% to 90% of its peak value, whereas the decay time *t*_*d*_ was defined as the time required for the pulse to decay from 90% to 10% of its peak value.

### Experimental Setup for Characterization under Pulsed MeV-Range X-Rays

Photoresponse under high-energy pulsed X-rays was investigated by means of a medical LINAC (Clinac iX, Varian). Acceleration voltage was set to 6 MV, allowing for the generation of photons with energy up to 6 MeV. Measurements were performed by positioning the detector at the LINAC isocenter and placing the device into a polymethylmethacrylate (PMMA) solid phantom at a depth of 5 cm and under a 10 × 10 cm^2^ field. A Keithley 6517A electrometer was used to acquire either the collected charge or the current signal of the detector during the irradiation (integration time 200 ms, sampling frequency 2 Hz). The bias voltage of the detector was set to 0 V. Experiments were remotely controlled by placing the instrumentation inside the shielded bunker room where the LINAC is located for patient treatment. Measurements were all performed under controlled temperature (22 °C) and humidity (40%), at a pressure of 1015 hPa, according to the radiotherapy treatment standards.

For charge measurements, the electrometer was set to record continuously the cumulative charge while the LINAC delivered a total nominal dose of 3 Gy at a dose rate of 50 mGy s^−1^. The effective dose (2.58 Gy) was measured with a FC 65-G (Farmer, IBA) ionization chamber connected to a Dose 1 electrometer (IBA). The ionization chamber was used under the same experimental conditions employed for the characterization of the device stack (LINAC isocenter, depth 5 cm, field size 10 × 10 cm^2^).

For current measurements, the electrometer was set to perform a relative acquisition, by automatically subtracting from readings the offset “dark” value measured before the start of X-ray irradiation. The dose rate ranged from 1 to 6 Gy min^−1^. The LINAC was set to deliver a nominal dose of 2 Gy (effective dose 1.72 Gy) at each dose rate. Average values and standard deviations of the current signals recorded during the “X-ray on” periods were used for the calculation of the photocurrent density as a function of the dose rate.

### Statistical Analysis

All statistical analyses were performed with Origin Pro 8 and Kaleidagraph 4.0 programs. All data were rounded to two significant digits after the decimal point. The data obtained from SEM, EDX, AFM, XRD, absorption spectroscopy, UV–Vis−NIR modulated photocurrent (amplitude and phase), *J-V*, dark current, and X-ray photocurrent were the original data without normalization. PL and TRPL data were normalized to unity. All the other data were obtained by transferring the corresponding original data according to the calculation formula.

Linear fittings were applied to the following plots: *SNR* vs. dose rate, mean photocurrent density (keV-range X-rays) vs. dose rate, Mott–Schottky plot, cumulative charge vs. delivered dose, mean photocurrent density (MeV-range X-rays) vs. dose rate. Power fittings were applied to the three segments of the SCLC plot obtained for the electron-only device (Fig. S26). Mono-exponential decay fitting was applied to TRPL and to photoconductive gain factor vs. absorbed photon flux (Figs. S18 and S19). Hecht’s fitting was applied to UV–Vis−NIR photocurrent amplitude vs. bias voltage obtained for the electron-only device.

The statistical distribution data of *PCE* (Fig. S4) were obtained from 123 independent devices.

## Results and Discussion

### Optical, Structural, and Morphological Properties of the Perovskite Active Layer

**Fig. 1:**
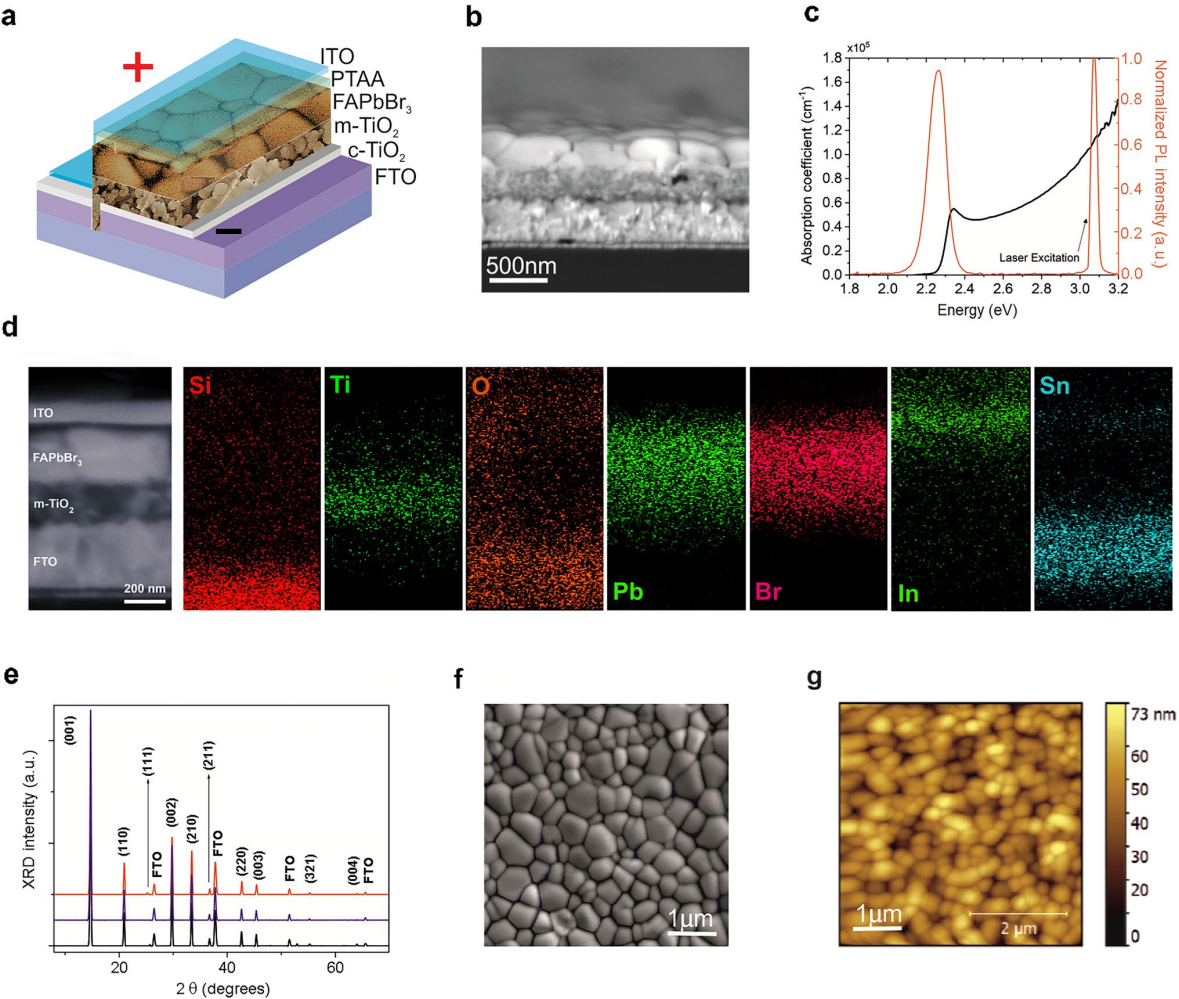
Device structure and basic material characterization. **a** Sketch of the complete device stack (Glass/FTO/c-TiO_2_/m-TiO_2_/FAPbBr_3_/PTAA/ITO) showing the electrode polarity used in the experiments with bias. **b** Cross-Sectional SEM image of the device stack before the deposition of the PTAA layer (Glass/FTO/c-TiO_2_/m-TiO_2_/FAPbBr_3_). **c** Absorption coefficient and photoluminescence spectra of the FAPbBr_3_ film. **d** EDX maps showing the elemental distribution of the complete device stack (Glass/FTO/c-TiO_2_/m-TiO_2_/FAPbBr_3_/PTAA/ITO). All maps have the same scale bar (200 nm) of the related cross-sectional SEM image shown on the left. **e** XRD patterns of the Glass/FTO/c-TiO_2_/m-TiO_2_/FAPbBr_3_ (black line), the Glass/FTO/c-TiO_2_/m-TiO_2_/FAPbBr_3_/PTAA (blue line), and the complete device Glass/FTO/c-TiO_2_/m-TiO_2_/FAPbBr_3_/PTAA/ITO (red line) stacks. Cubic FAPbBr_3_ Miller indexes are also shown. FTO reflections are labeled accordingly to crystallographic database ICDD card No. 00–001-0657. **f** Planar SEM image of the FAPbBr_3_ film after the deposition by solvent quenching method. **g** AFM image (5 × 5 μm^2^) of the FAPbBr_3_ film surface

The scheme of the fabricated perovskite X-ray detector is represented in Fig. 1a. The device stack is the following: FTO/c-TiO_2_/m-TiO_2_/FAPbBr_3_/PTAA/ITO. The FAPbBr_3_ perovskite film is the active layer, *i.e.*, it converts the absorbed X-ray photons into electric charge. Compact TiO_2_ (c-TiO_2_) and PTAA layers extract the photogenerated electrons and holes, respectively, from the FAPbBr_3_ active layer and then transfer them to the FTO and ITO electrodes. The FAPbBr_3_ film is deposited by solvent quenching method onto a mesoporous TiO_2_ (m-TiO_2_) layer, which therefore serves both as an electron transport layer and as a scaffold. The thickness of the m-TiO_2_ layer and the FAPbBr_3_ over-layer, as inferred from cross-sectional SEM images of the device stack before the deposition of the PTAA layer (Fig. 1b), are 245 ± 5 and 255 ± 5 nm, respectively. The absorption coefficient, as measured from the absorbance spectrum shown in Fig. 1c (black curve), returns a band gap of 2.28 eV. The PL spectrum (Fig. 1c, red curve) shows an emission peak at 2.27 eV, with a narrow FWHM (full width at half maximum) of about 0.11 eV. The PL stability was evaluated by measuring the PL signal for 6 min, without showing a significant decrease in intensity (Fig. S1). Elemental maps, obtained by EDX spectroscopy to assess the composition of the different layers forming the device stack, are shown in Fig. 1d; as can be seen, the Pb and Br maps confirm the presence of a dense FAPbBr_3_ layer deposited onto the mesoporous TiO_2_ scaffold. In addition, as can be inferred from Fig. S2, reporting the EDX spectrum recorded from the complete device stack in the 0 – 10 keV range, the [Br]/[Pb] concentration ratio is 3.27, denoting a correct stoichiometry of the FAPbBr_3_ active layer. The complete device stack (FTO/c-TiO_2_/m-TiO_2_/FAPbBr_3_/PTAA/ITO) and the intermediate samples (FTO/c-TiO_2_/m-TiO_2_/FAPbBr_3_/PTAA) were characterized by XRD, in order to obtain structural information on the crystalline structure of the different layers composing the detectors, the so obtained diffraction patterns being reported in Fig. 1e. As can be seen, the perovskite diffraction patterns show the exclusive presence of polycrystalline cubic FAPbBr_3_ phase, space group *Pm3m*, consistently with previously reported results [37, 38]. Finally, the morphological properties of the polycrystalline FAPbBr_3_ layer were characterized by planar SEM (Fig. 1f) and AFM (Fig. 1g), aimed at evaluating the perovskite grain size and roughness, respectively. The SEM image shows the presence of large crystal grains with size up to 1 μm and complete surface coverage. The AFM image reveals an ultra-flat layer with RMS (root mean square) roughness lower than 15 nm.

### Device Reproducibility

Aimed at building a robust statistics on the device reproducibility, 123 samples (some of them are shown in Fig. S3) were fabricated and tested as solar cells by measuring the current density to voltage (*J-V*) characteristics and the power conversion efficiency (*PCE*) at maximum power point (MPP). As can be seen from Fig. S4, the 96.75% of the tested devices show a *PCE* in the 5.75%–7.75% range in the case of forward scan direction (6.70% on the average), highlighting a very good device reproducibility. The *J-V* characteristics of the best-performing device are reported in Fig. S5, showing a remarkably high open-circuit voltage (*V*_oc_) value of 1.54 V. We would like to stress here that, although the aim of this work is not the fabrication of semi-transparent perovskite solar cells (ST-PSCs), the device stack under test represents the state-of-the-art for the ST-PSC field [39, 40], returning an average visible transmittance (*AVT*) of 52% (Fig. S6), a *PCE* of 8.20% (Fig. S7a) and a light utilization efficiency (*LUE*) of 4.30% (Fig. S7b).

### Photoelectronic Properties in the UV–Vis–NIR Range

Two samples (indicated with FS-VI and FS-VIII) were then randomly selected and tested for a deeper investigation of the photoelectronic properties of the device stack in the UV–Vis–NIR wavelength range. Modulated photocurrent measurements further confirm the good device reproducibility, as can be inferred from both the amplitude (Fig. S8) and phase (Fig. S9) spectra recorded for the two samples in the 200–700 nm wavelength range at a 0 V bias voltage (*V*_B_), which are almost perfectly overlapping. In both cases, the amplitude spectrum does not show significant features for wavelengths (*λ*) higher than 600 nm, denoting a negligible concentration of electrically active defects: this is confirmed by the phase spectrum, showing a completely unlocked signal for *λ* > 600 nm.

**Fig. 2:**
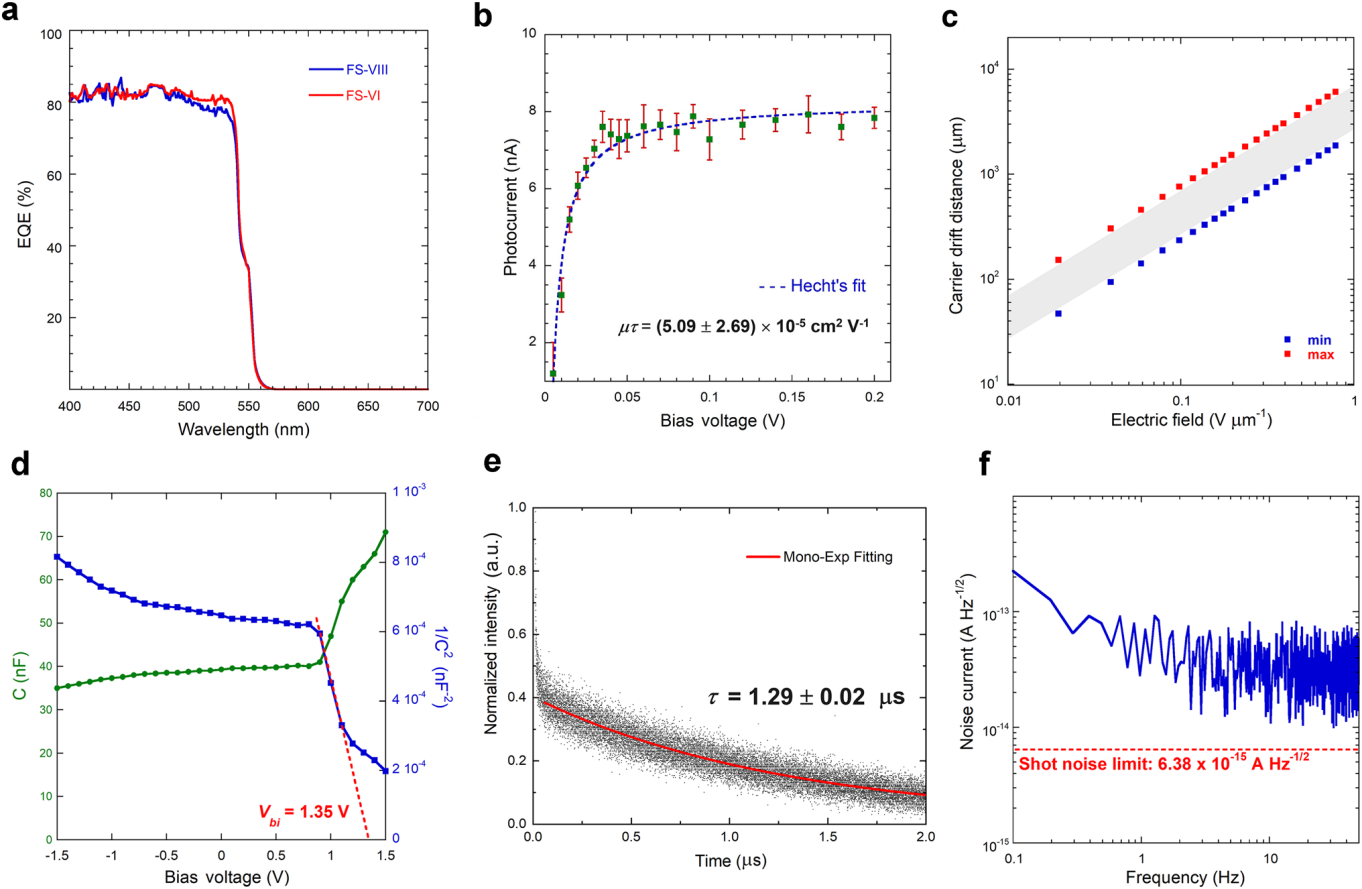
Photoelectronic properties in the UV–Vis–NIR range. **a** External quantum efficiency of two different FAPbBr_3_ complete devices (FS-VI and FS-VIII) in the 400–700 nm wavelength range at *V*_B_ = 0 V. **b** Amplitude of the modulated photocurrent (*I*_*ph*_) under monochromatic light (*λ* = 375 nm) of a FAPbBr_3_ electron-only device as a function of the applied bias voltage (*V*_B_). Dashed blue line indicates the best fit to data obtained by using Hecht's equation (see Sect. 2.5 for details). **c** Carrier drift distance (*L*) of the FAPbBr_3_ electron-only device as a function of the applied electric field *E*_B_ = *V*_B_/*d*. Minimum (blue dots) and maximum (red dots) values were calculated from the minimum and maximum values obtained for the *µ*_*e*_*τ*_*e*_ product. Gray box is a visual guide to indicate all the possible values of *L*. **d** Capacitance–voltage plot (green) and Mott–Schottky plot (blue) of a FAPbBr_3_ complete device. Red dotted line indicates the best linear fit to data corresponding to the linear region of the Mott–Schottky plot. The X-intercept of the red dotted line returns the built-in potential (*V*_*bi*_ = 1.35 V). **e** Normalized photoluminescence decay under monochromatic light (*λ* = 375 nm) of a FAPbBr_3_ electron-only device. Red solid line indicates the best fit to data obtained by using a mono-exponential decay equation. **f** Noise current spectrum of a FAPbBr_3_ complete device in the 0.1–50 Hz range. The average value (38 fA Hz^−1/2^) was used to estimate the device detectivity. Red dashed line indicates the limit of the shot noise

From the current amplitude spectra, the most significant figures of merit of a photodetector can be derived, namely responsivity (Fig. S10), external quantum efficiency (Fig. 2a), and specific detectivity (Fig. S11). Responsivity (*R*) is higher than 0.3 A W^−1^ in the 450–550 nm range, reaching its maximum value (0.35 A W^−1^) at the bandgap wavelength (*λ*_*g*_ = 543 nm). External quantum efficiency (*EQE*), evaluated in the same wavelength range, is constantly higher than 80%. It is worth mentioning here that both *R* and *EQE* values are significantly higher than those recently reported on FA-based perovskites proposed for X-ray detection, all below 0.15 A W^−1^ and 20%, respectively [20, 23, 24], denoting the exceptionally high charge collection efficiency of the proposed device. This was confirmed after the investigation on the main charge transport properties of the active layer, performed on a dedicated electron-only device (Glass/FTO/c-TiO_2_/m-TiO_2_/FAPbBr_3_/ITO). As can be seen from Fig. 2b, reporting the modulated photocurrent under UV monochromatic light (*λ* = 375 nm) as a function of the applied bias voltage, signal saturation (implying a 100% charge collection efficiency) is already obtained at *V*_B_ = 0.04 V, and the estimated electron mobility–lifetime product is *µ*_*e*_*τ*_*e*_  = (5.09 ± 2.69) × 10^–5^ cm^2^ V^−1^, comparable to the highest values reported on submicrometer-thick MHP polycrystalline films for X-ray detection [41]. From the obtained *µ*_*e*_*τ*_*e*_ value, it is possible to estimate the drift distance of the photogenerated electrons, defined as *L* =* µ*_*e*_*τ*_*e*_*F*_B_, where *F*_B_ = *V*_B_*/d* is the applied electric field. As can be seen from Fig. 2c, *L* ranges from about 50 μm (the minimum value at *F*_B_ = 0.02 V µm^−1^) to about 6 mm (the maximum value at *F*_B_ = 0.78 V µm^−1^), which are far higher values than the thickness of the active layer (255 nm). Therefore, by considering that the bias voltage *V*_B_ plays in the electron-only device the role of the built-in potential responsible for charge drifting in the self-powered complete device stack, we can infer that the photogenerated charge carriers can be effectively collected by the electrodes. For an accurate determination of the built-in potential *V*_*bi*_, the capacitance (*C*) of a complete device stack, selected from the same batch described in Sect. 3.2, was measured as a function of the applied bias voltage. Results are reported in Fig. 2d. As can be inferred from the 1/*C*^2^ vs. *V*_B_ curve (Mott–Schottky plot), a built-in potential *V*_*bi*_ = 1.35 V can be estimated, highlighting the presence of a very intense internal electric field (*F*_*in*_ = *V*_*bi*_/*d* = 5.3 V µm^−1^).

Time-resolved photoluminescence (TRPL) measurements, performed on the electron-only device at the same wavelength (*λ* = 375 nm) as the modulated photocurrent measurements shown in Fig. 2b, point out a decay time *τ* = 1.29 ± 0.02 μs (Fig. 2e). From the value of the *µ*_*e*_*τ*_*e*_ product, it is then possible to estimate an electron mobility equal to *µ*_*e*_  = 39.4 ± 20.9 cm^2^ V^−1^ s^−1^, consistently with the values reported on FAPbBr_3_ thin films [42], and highlighting the excellent charge transport properties of this material even in the polycrystalline form. Of course, this is only a rough estimation, being obtained by combining transient photoluminescence to steady-state phototransport results. In addition, it is known that the decay time obtained from TRPL measurements is related to minority carriers, so it is not possible, being the FAPbBr_3_ layer undoped, to determine which type of carrier contributes to the TRPL signal. However, we have to consider that electrons and holes have been demonstrated to follow approximately the same transport dynamics in FAPbBr_3_: mobility values are indeed in the same range (on the order of a few tens of cm^2^ V^−1^ s^−1^) [43, 44], so it is reasonable to suppose that, in case of an undoped material, a similar concentration of recombination centers is present for electrons and holes, implying similar carrier lifetimes.

The specific detectivity (D*) exceeds 1 × 10^12^ cm Hz^1/2^ W^−1^ in the full 350–550 nm wavelength range, reaching peak values at the bandgap wavelength of 5.8 × 10^12^ cm Hz^1/2 ^W^−1^ and 4.9 × 10^12^ cm Hz^1/2^ W^−1^ for the FS-VI and FS-VIII devices, respectively, comparable to the D* value (3.5 × 10^12^ cm Hz^1/2^ W^−1^) recently reported on X-ray and visible light detectors based on single-crystal FAPbBr_3_ [21]. The high detectivity reflects the ability of the device to detect very weak light intensity and is strictly related to the very low noise current measured in photovoltaic mode (Fig. 2f).

The dark current density (*J*_*d*_) vs. bias voltage characteristics of FS-VI and FS-VIII devices (Fig. S12) show that *J*_*d*_ is well below 0.5 nA cm^−2^ at 0 V, but it increases of about 2 orders of magnitude when applying a bias voltage of only ± 0.5 V (*i.e*., both in the forward and reverse bias mode), upshifting the baseline noise of the modulated photocurrent amplitude (Fig. S13). Dark current is not only much lower at *V*_B_ = 0 V, but significantly more stable over time: the application of even a small bias voltage (*e.g*., - 0.1 V) implies indeed a significant dark current drift, as well as a larger standard deviation (Fig. S14). This is most probably caused by ion migration, which has been recently demonstrated to account for the instability of detectors based on lead-halide perovskites [45]. All of these considerations, along with the intrinsic advantage of operating at *V*_B_ = 0 V, reinforced our choice to perform experiments under X-ray irradiation in self-powered mode.

### Evaluation of the Detection Performance under keV-Range X-Rays

**Fig. 3:**
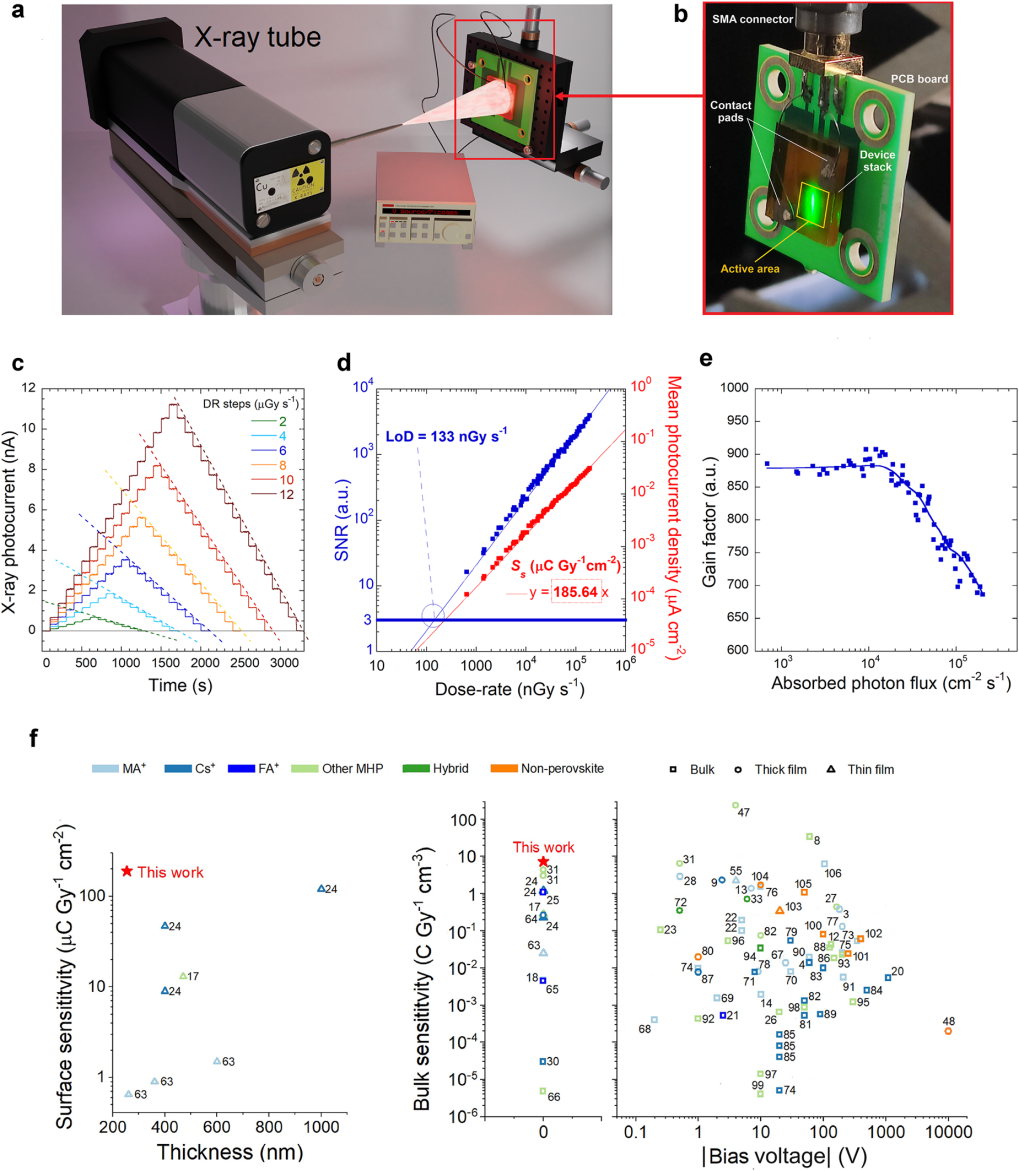
Evaluation of the detection performance under keV-range X-rays. **a** Sketch of the keV-range X-ray irradiation setup. The detector is mounted on a *xyz* stage used for the automatic centering procedure of the X-ray beam spot. **b** Picture of the prototypal device under test. The picture was taken during UV–Vis–NIR photoconductivity measurements (the green spot is the monochromator output at *λ* = 555 nm), but the same device configuration was used for X-ray tests. **c** X-ray photocurrent as a function of time recorded at different radiation dose rate steps. Dashed lines are a guide to the eye to highlight the response linearity with dose rate. **d** Signal-to-noise ratio (blue squares) and mean X-ray photocurrent density (red squares) as a function of dose rate. Blue and red thin lines indicate the best linear fits to the experimental data. The limit of detection (*LoD*) is obtained from the intercept (blue circle) of the blue fitting line with *SNR* = 3 (thick blue line). The surface sensitivity is obtained from the slope of the red fitting line. **e** Photoconductive gain factor (*G*) as a function of the absorbed photon flux (*φ*) at a photon energy *E*_*ph*_ = 8.05 keV. The blue continuous line is a visual guide. **f** Surface specific sensitivity vs. thickness of self-powered thin-film-based direct X-ray detectors (left) and bulk specific sensitivity vs. applied bias voltage (in absolute value) of state-of-the-art solid-state photoconductors and photodiodes for “hard” X-rays (right) reported in the literature [3, 4, 8, 9, 12–14, 17, 18, 20–28, 30, 31, 33, 47, 48, 55, 63–106] . The legend labels “MA^+”^, “Cs^+^” and “FA^+^” are used for the three most common single-cation MHPs. “Other MHP” include mixed-cation MHPs and other single-cation MHPs employing cations different from MA^+^, Cs^+^, and FA^+^. The term “hybrid” indicates devices based on hybrid MHP/non-perovskite active layers. The term “bulk” is used for free-standing active bulk devices. “Thick film” and “Thin film” indicate devices based on thick (> 10 μm) and thin (≤ 10 μm) films deposited on free-standing non-active substrates, respectively

The sample FS-VI, showing an average dark current of only 127 pA, was selected for extensive characterization under a continuous 40 kV X-ray beam (Fig. 3a, b) produced by a Cu target X-ray tube. The first features to highlight (Fig. S15) are the fast response (rise time < 0.5 s), the instantaneous settlement of the X-ray photocurrent signal to a stationary value, and the quick recovery time after the end of an X-ray irradiation period (decay time < 0.25 s). All of these properties are essential for monitoring reliably the intensity of an X-ray source, which is crucial for medical applications such as interventional radiology or radiation therapy for cancer treatment, where the device can be effectively used for accurate real-time dosimetry.

The detector also demonstrated to produce a photocurrent able to instantaneously follow the variations of the X-ray dose rate with time (Fig. 3c), returning stable and reproducible values regardless of the amplitude of the dose rate steps. Most significantly, as can be seen from Fig. 3d (red plot), the X-ray photocurrent density is linear with the dose rate in the full measured range (644 nGy s^−1^–189.3 μGy s^−1^). From the slope of the curve, the surface specific sensitivity is evaluated to be *S*_*s*_ = 185.64 μC Gy^−1^ cm^−2^, which is the highest value reported on self-powered thin-film-based (*i.e*., with active layer thickness < 10 μm) direct X-ray detectors (Fig. 3f, left). It is noteworthy to emphasize that, considering the thickness of the FAPbBr_3_ active layer (255 ± 5 nm), the calculated bulk specific sensitivity (*i.e*., the sensitivity normalized to the active volume) amounts to *S*_*v*_ = 7.28 ± 0.14 C Gy^−1^ cm^−3^. If we consider the whole range of solid-state photoconductors and photodiodes for “hard” X-rays (*i.e*., with energy conventionally > 2 keV) [46], either perovskite-based or not, including not self-powered detectors (Fig. 3f, right), this is the highest value ever reported for thin-film-based devices. It is only exceeded by bulk FAMACs single crystals [8], however requiring a high bias voltage (100 V) to operate, and by quasi-monocrystalline MA_0.42_FA_0.58_PbI_3_ thick films [47], showing outstanding sensitivity at the expenses of response speed and signal stability over time. It is finally worth highlighting that the obtained *S*_*v*_ value surpasses that of amorphous Se, the reference material for the production of commercial X-ray flat-panel detectors employed in digital radiography, by more than four orders of magnitude [48]. This remarkable achievement comes with the undeniable advantage of not necessitating a bias voltage for operational purposes.

The limit of detection (*LoD*) is another important figure of merit of an X-ray detector, because it is strictly related to health risk during X-ray inspection. According to the IUPAC (International Union of Pure and Applied Chemistry) definition, the *LoD* represents the lowest dose rate at which the signal-to-noise ratio (*SNR*) is equal to 3. In the case of our device, the *LoD* is evaluated to be 133 nGy s^−1^ (Fig. 3d, blue plot), which is comparable to that reported for FAPbBr_3_ single-crystal X-ray detectors, but significantly lower than the minimum *LoD* required for medical diagnostics (5.5 μGy s^−1^) [49]. The estimated *LoD* value is also experimentally validated by irradiating the device at 644 nGy s^−1^, *i.e.,* the minimum dose rate obtainable with our X-ray tube (Fig. S16).

It is worth elaborating here on the possible reasons that lie behind the outstanding sensitivity of our detector, by exploring the relationship between the measured X-ray photocurrent and the photon absorption capabilities of the active layer. As somewhat expected from a submicrometer-thick active layer, the surface specific sensitivity we found experimentally (*S*_*s*_ = 185.64 μC Gy^−1^ cm^−2^) exceeds the upper theoretical limit *S*_*max*_ = 0.23 μC Gy^−1^ cm^−2^ (see Supplementary Note S1) by almost three orders of magnitude. This is, however, not surprising on the basis on the very recent literature on MHP-based thin films for X-ray detection, in which the reported sensitivity often exceeds the theoretical limit [31], even of four orders of magnitude [50]. The relation *S*_*s*_ > *S*_*max*_ at a specific X-ray energy *E*_*ph*_ can only be explained by the presence of: 1) a significant signal contribution given by photons with energy *E* < *E*_*ph*_, such as those of the *Bremsstrahlung* radiation produced by an X-ray tube, which can be more easily absorbed by the active layer; 2) an internal photoconductive gain occurring when *E* = *E*_*ph*_. However, in the case of commercial Cu target X-ray tubes for diffractometry, as the one used in this work, the *Bremsstrahlung* radiation produced in the 1–8.05 keV range (*i.e.* with energy *E* < *E*_*ph*_ = 8.05 keV, considered as the most probable energy for the calculation of *S*_*max*_) accounts for less than 1% of the total emitted radiation, and is also more efficiently absorbed in air. As a consequence, the primary factor of the sensitivity enhancement must be photoconductive gain.

It is known that photoconductive gain is obtained when the recombination lifetime of the photogenerated charge carriers is longer than their transit time [51] across the active layer, allowing for carrier accumulation and leading to an effective charge multiplication. In our case, we hypothesize that photoconductive gain is assisted by defect-related traps, capturing charge carriers and prolonging their recombination lifetime. To perform a quantitative analysis, trap density *n*_*t*_ was estimated by space-charge-limited-current (SCLC) technique for an electron-only device (see Supplementary Note S2), returning *n*_*t*_ = (1.54 ± 0.06) × 10^15^ cm^−3^. However, the only presence of a significant distribution of traps is not sufficient to ensure photoconductive gain. Two other conditions must be met: 1) a high internal electric field, able to promote an efficient de-trapping of trapped carriers; 2) an absorbed photon flux (*φ*) low enough to avoid filling up of the trap levels. In all the experiments performed in this work, either under X-rays or UV–Vis–NIR light irradiation, the first condition was always met. Despite the externally applied bias voltage is 0 V, the internal electric field can be indeed very intense: indeed, as estimated in Sect. 3.3, *F*_*in*_ can easily reach values in the V µm^−1^ range_,_ which is high enough to enable carrier de-trapping and trigger photoconductive gain [31]. It is worth recalling here that this is the typical range of externally applied electric field in which purely photoconductive semiconductor-based detectors for ionizing radiation reach a 100% charge collection efficiency (*CCE*), regardless of their thickness [52]. As for the second condition, it was met only under X-rays, where the very low absorbed photon flux, evaluated to be in the *φ* = 10^3^–10^5^ cm^−2^ s^−1^ range, implies a photogenerated charge density always significantly lower than the trap density; conversely, under UV–Vis–NIR light irradiation, the photon flux of the lamp used for the spectral photoconductivity experiments was high enough to rapidly fill up the trap levels responsible for the photoconductive gain, and this explains why the measured *EQE* (Fig. 2a) is below 100%, (*i.e.,* no gain is observed).

To further support such conclusions, it is worth observing the trend of the gain factor *G* = *J*_*ph*_*/J*_*max*_*,* defined as the ratio between the measured and theoretical photocurrent density, as a function of the absorbed photon flux under X-rays (Fig. 3e): after assuming a constant value at low fluxes (in the *φ* = 10^3^–10^4^ cm^−2^ s^−1^ range), *G* starts decreasing exponentially with increasing *φ* values (Fig. S18). This can be understood by supposing that, when the density of photogenerated charge carriers is very low, there is a substantial balance between trapping and de-trapping (*i.e*., between filled and emptied trap levels), so that the contribution of trap-assisted recombination to the photocurrent signal, which is responsible for the gain, is constant with *φ.* When the absorbed photon flux increases over 10^4^ cm^−2^ s^−1^, trap levels are gradually filled up, making band-to-band recombination more and more dominant on trap-assisted recombination, thus gradually quenching photoconductive gain. By extrapolating data up to the absorbed photon flux necessary to have *G* = 1 (Fig. S19), we obtain *φ* = 4.25 × 10^6^ cm^−2^ s^−1^, which is far lower, as expected, than the photon flux used for experiments under UV–Vis radiation (always in the 10^13^–10^14^ cm^−2^ s^−1^ range, depending on the lamp output power at the different wavelengths).

Even if based on a rough quantitative analysis, these results strengthen our hypothesis on the dominant role played by traps in extending the device sensitivity beyond the theoretical limit. More significantly, it is shown that the presence of traps for charge carriers may not necessarily be detrimental to the performance of an X-ray detector operating in current-mode, but may even be essential for a submicrometer-thick device, because it overcompensates for its intrinsically low X-ray photon absorption capability. The contribution of the thickness of the active layer to increase the sensitivity of the device is indeed limited: suffice it to say that, even by supposing a film thickness of 1 μm, which can be approximately considered as the upper technological limit for MHP films obtainable by spin coating, the relative X-ray absorption at 8 keV (Fig. S20) is only 5.33%, resulting in *S*_*max*_ = 0.9 μC Gy^−1^ cm^−2^, which is still more than two orders of magnitude lower than the experimental surface sensitivity found with the 255 nm-thick film investigated in this work. Moreover, it is worth observing here that a higher thickness would imply a weaker built-in electric field in the active layer, possibly decreasing the trap-assisted photoconductive gain.

### Long-Term Operational Stability Tests under X-Ray Irradiation

**Fig. 4:**
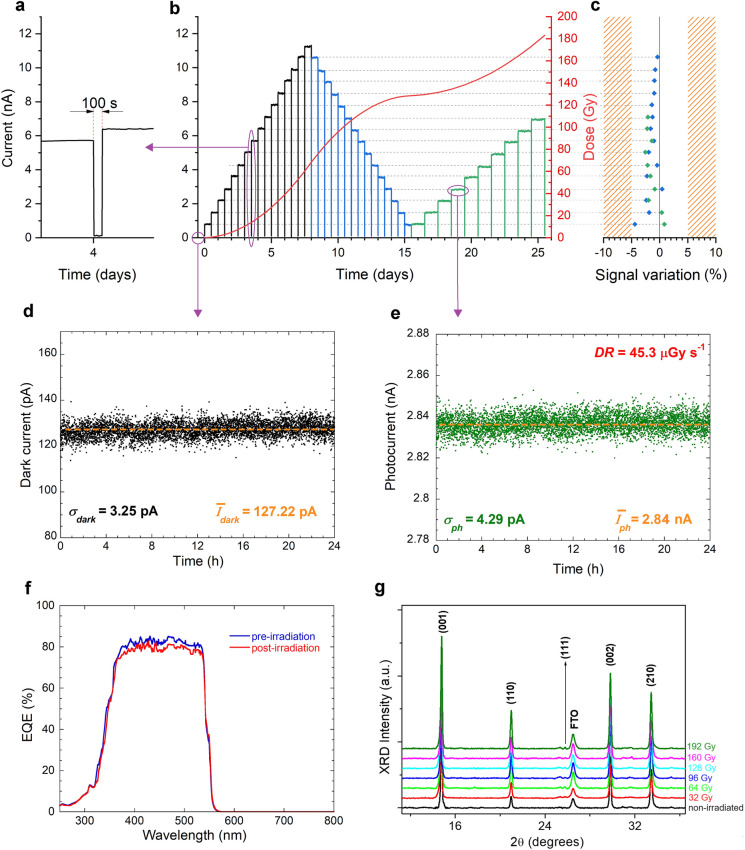
Long-term operational stability tests under X-ray irradiation. **a** Zoomed image of the current recorded during the 100 s “X-ray off” period between two consecutive 12-h long “X-ray on” periods. **b** Current measured during the whole 26-day long irradiation period, structured as the following: dose rate increasing every 12 h (black), dose rate decreasing every 12 h (blue), and dose rate increasing every 24 h (green). Dose rate was always varied by steps of 12 μGy s^−1^, with a minimum value of 9.3 μGy s^−1^ and a maximum value of 189.3 μGy s^−1^. Red curve shows the accumulation of the ionizing dose during the 26 days. **c** Variation of the photocurrent signal between two “X-ray on” periods at the same dose rate; blue diamonds refer to variations between the first and the second cycle; green diamonds refer to variations between the first and the third cycle. Dashed lines are a guide to the eye to indicate signals at equal dose rates. **d** Dark current recorded within a 24-h long period before X-ray irradiation. **e** X-ray photocurrent recorded within a 24-h long irradiation period at a dose rate of 45.3 μGy s^−1^. **f** External quantum efficiency in the 250–800 nm wavelength range evaluated before and after the 26-day long irradiation period at *V*_B_ = 0 V. **g**. XRD patterns of a Glass/FTO/c-TiO_2_/m-TiO_2_/FAPbBr_3_/PTAA/ITO stack (complete device) recorded after exposure to increasing values of total ionizing dose (*TID*) in the 32–192 Gy range

After the assessment of the performance in terms of response speed, linearity with radiation dose rate, sensitivity and limit of detection, the prototypal device FS-VI was subjected to a 26-day long continuous irradiation under X-rays. Results are reported in Fig. 4. Before irradiation, a 24-h long dark current measurement was performed (Fig. 4d). Dark current settled immediately to an average value of about 127 pA, which was maintained unaltered for 24 h with a standard deviation of only 3.25 pA. Moreover, no appreciable dark current drift, which has often been reported as one of the major drawbacks of perovskite-based detectors for ionizing radiation [6, 13–15, 53], was observed within the measurement time, pointing out an exceptionally high stability.

The detector was subsequently tested under three different cycles of X-ray uninterrupted irradiation for a total of 26 days (Fig. 4b). Cycles were structured as the following: in the first cycle, dose rate was increased every 12 h; in the second cycle, dose rate was decreased every 12 h; in the third cycle, dose rate was increased every 24 h. At each variation of the dose rate, X-rays were shut down for only 100 s (Fig. 4a), which is however a time long enough to reset completely the device, with no presence of persistent photocurrent. The first outstanding result to point out is that signals recorded at the different dose rates do not show any temporary positive or negative drift from their average values within a single 24-h long irradiation period (Fig. 4e). This is an unprecedented result in the field of detectors exploiting MHPs as active material, improving that obtained with PFMs [33]. Moreover, as can be seen from Fig. 4b, reporting the X-ray photocurrent values measured during the whole 26-day measurement session, the signal corresponding to a given dose rate is not only stable within a single 12-h or 24-h long period, but it is also perfectly reproducible from one cycle to the other, even after several days of uninterrupted irradiation. The signal variations from the initial values (Fig. 4c) after long-term irradiation span from  -4.5% to + 1%, with an average value of only -1.3%. It is worth highlighting here that, up to now, the operational stability of self-powered X-ray detectors based on perovskite films has been assessed for no longer than 1 h under continuous irradiation [24], or 1600 s by successively tuning on and off the X-ray source [31], showing in both cases a slight loss of sensitivity.

At the end of the 26-day long X-ray irradiation period, the prototypal device was again tested under UV–Vis–NIR radiation, aimed at verifying the presence of electrically active defects possibly caused by radiation damage, which are responsible for the introduction of allowed energy levels (or bands) within the material bandgap. Very remarkably, no sub-bandgap features (*i.e*., for *λ* > *λ*_*g*_) can be observed in the *EQE* spectrum recorded after the long-term irradiation (Fig. 4f), substantially denoting the structural integrity of the whole stack and the absence of a significant radiation-induced damage. Moreover, the two *EQE* spectra (before and after the irradiation) are almost coincident for *λ* < *λ*_*g*_, highlighting neither significant degradation of the photoelectronic properties (thus implying the stability of the active material) nor presence of additional electrically active defects. The average *EQE* evaluated after irradiation in the 400–550 nm wavelength range is 79.4%, which is only 2.8% lower than that measured before irradiation (82.2%). Responsivity behaves similarly (Fig. S21), being still higher than 0.3 A W^−1^ around *λ*_*g*_ even after the prolonged exposure to X-rays. As regards D* (Fig. S22), the performance degradation, even if limited, is higher than *EQE* and *R*: the specific detectivity in the 400–550 nm wavelength range decreases indeed by 5.8%, most probably due to a slight increase of the dark current, as measured at the end of the irradiation period, when the X-ray tube was definitively switched off (Fig. S23).

As can be inferred from the evaluation of the photoelectronic properties after the prolonged irradiation, our prototypal device demonstrated an excellent radiation hardness: this is quite unexpected for metal-halide perovskite thin films, which usually report structural damage after few hours of exposure to X-rays in air [54]. In a subsequent phase of our study, aimed at a deeper investigation of the radiation hardness of the FAPbBr_3_ film, a complete device stack selected from the same batch described in Sect. 3.2 was exposed to the same total ionizing dose (*TID*) as the sample used for the 26-day long test (192 Gy, red curve of Fig. 4b). Several XRD measurements (Fig. 4g) were progressively performed during the experiment in order to monitor the structural integrity of the active layer: as can be seen, despite the high accumulated X-ray dose, the cubic structure of FAPbBr_3_ is perfectly preserved, and no signs of structural degradation/modification is observed. It is worth highlighting here that a *TID* value of 192 Gy exceeds of about two orders of magnitude the highest *TID* reported up to now (2.2 Gy) on MHP thin films [55]. In the literature on MHP-based X-ray detectors, comparable values of *TID* have only been reported for millimeter-thick single crystals [56, 91] or very thick (hundreds of micrometers) polycrystalline films [67], but have been obtained by exposing the devices to high dose rates for short times, mostly aimed at evaluating the radiation hardness. However, a good radiation hardness is a necessary but not sufficient condition for a reliable X-ray detector, and this explains the necessity of a prolonged test. Monitoring the photocurrent signal under uninterrupted irradiation for a considerable amount of time (*e.g*., weeks), so to take into account the possible effects of temperature and humidity on the performance of the detector, is indeed the most accurate method to assess the signal stability and reproducibility over time. And this is even more crucial for perovskite-based devices, the photoelectronic properties of which may be very sensitive to environmental conditions. In addition, the 26-day long test gave us the opportunity to assess the operational stability of the detector even at very low dose rates, as those used in the field of interventional radiology: in this case, signals may indeed be significantly weak, and the presence of instability over time (due, for instance, to noise drift) would be more obvious. It is also worth noting here that the signal stability and reproducibility, as well as the negligible degradation of the optoelectronic properties, are sufficient conditions to classify our detector at least as a rad-hard “D-level” (*i.e.*, compliant with a *TID* > 100 Gy) device according to US MIL-PRF-38535F, which is the reference standard for electronic and optoelectronic devices suitable for military and space applications. Obviously, the upper *TID* limit of the device (*i.e.,* the *TID* corresponding to unacceptable performance degradation) is significantly higher, and depends on the “acceptable tolerance levels” (*ATL*) defined for a specific dosimeter operating in a specific field of application. For instance, the *ATL* established by *IEC* (International Electrotechnical Commission) for X-ray dosimeters used in interventional radiology [57] is ± 25%. This allows us to make a rough prediction of the operating lifetime of the device for this specific application. By considering that -1.3% is the average signal loss after exposure to a *TID* = 192 Gy, and assuming a linear degradation model with an acceptable performance degradation of -25%, we can predict that our device can operate up to a *TID* = 3.7 kGy. Of course, this is only a rough extrapolation, which does not take into account other possible degradation factors in addition to radiation-induced damage (*e.g*., environmental factors), but highlights anyway the potentially excellent radiation hardness of the device: just to give an example, a *TID* = 3.7 kGy corresponds to the total X-ray dose delivered in about 12,600 pulmonary angiographies [58], that is one of the routine procedures in interventional radiology.

At the molecular level, we speculate that self-healing of the active layer, reported to occur only *in vacuo* for FAPbBr_3_ films [54, 59], is the primary responsible for the stable performance of our device even under X-ray irradiation at atmospheric conditions, as well as of its radiation hardness. As reported in a very recent study [59] on the modification of chemical and optoelectronic properties of FAPbBr_3_ thin films (grown with the same method as described in this work) under X-rays in vacuum conditions, FAPbBr_3_ decomposes as expected into FABr and PbBr_2_, with the latter decomposing further into metallic lead (Pb^0^) and Br_2_ gas. This would unavoidably result in the permanent degradation of perovskite, with formation of Pb^0^ metallic clusters and loss of Br_2_ from the surface. However, in the bulk material, degradation is followed by a self-healing process through re-oxidation of Pb^0^ and enhanced migration of FA^+^ and Br^−^ ions, which favors the capture of gaseous Br_2_ as a reagent, thus reforming perovskite and recovering its optoelectronic properties. We hypothesize that the same degradation/self-healing “circular” process is most likely triggered also at atmospheric pressure, because it pertains to the bulk material, and not to the surface, from which obvious degassing of Br_2_ would occur. It is worth observing that, in the case of our device stack, the possible loss of Br_2_ is further limited by the presence of the PTAA film over the active layer. In addition, the XRD experiments performed after exposure to progressively increasing X-ray doses (Fig. 4g), showing that the FAPbBr_3_ structure is not modified by the absorbed radiation, directly indicate that no loss of Br_2_ occurs from the surface: this further supports the hypothesis of a degradation/self-healing mechanism.

It is also possible to phenomenologically demonstrate that mesoporous TiO_2_ is essential to the stability over time of the X-ray photoresponse, as well to improve the signal-to-noise ratio. Indeed, we compared the performance of our device stack to that of another stack fabricated without the m-TiO_2_ scaffold (*i.e.*, Glass/FTO/c-TiO_2_/FAPbBr_3_/PTAA/ITO). As expected, the sensitivity of the two devices, being mostly due to the photoconductive properties of the FAPbBr_3_ active layer, is approximately the same. But, as can be seen from Fig. S24, the dark current is higher without the m-TiO_2_ layer, and a positive drift is observed; as a consequence, the limit of detection increases, and the stability over time of the X-ray photoresponse is negatively affected (Fig. S25). This increase in dark current is consistent with the hypothesis of moisture penetration within the device, implying a slight decrease in dark resistivity. Indeed, m-TiO_2_ scaffolds have been reported to act as barriers to moisture [60], thus protecting the active layer and contributing to the stability over time of the device performance.

### Validation of the X-Ray Detector in Relevant Environment

**Fig. 5:**
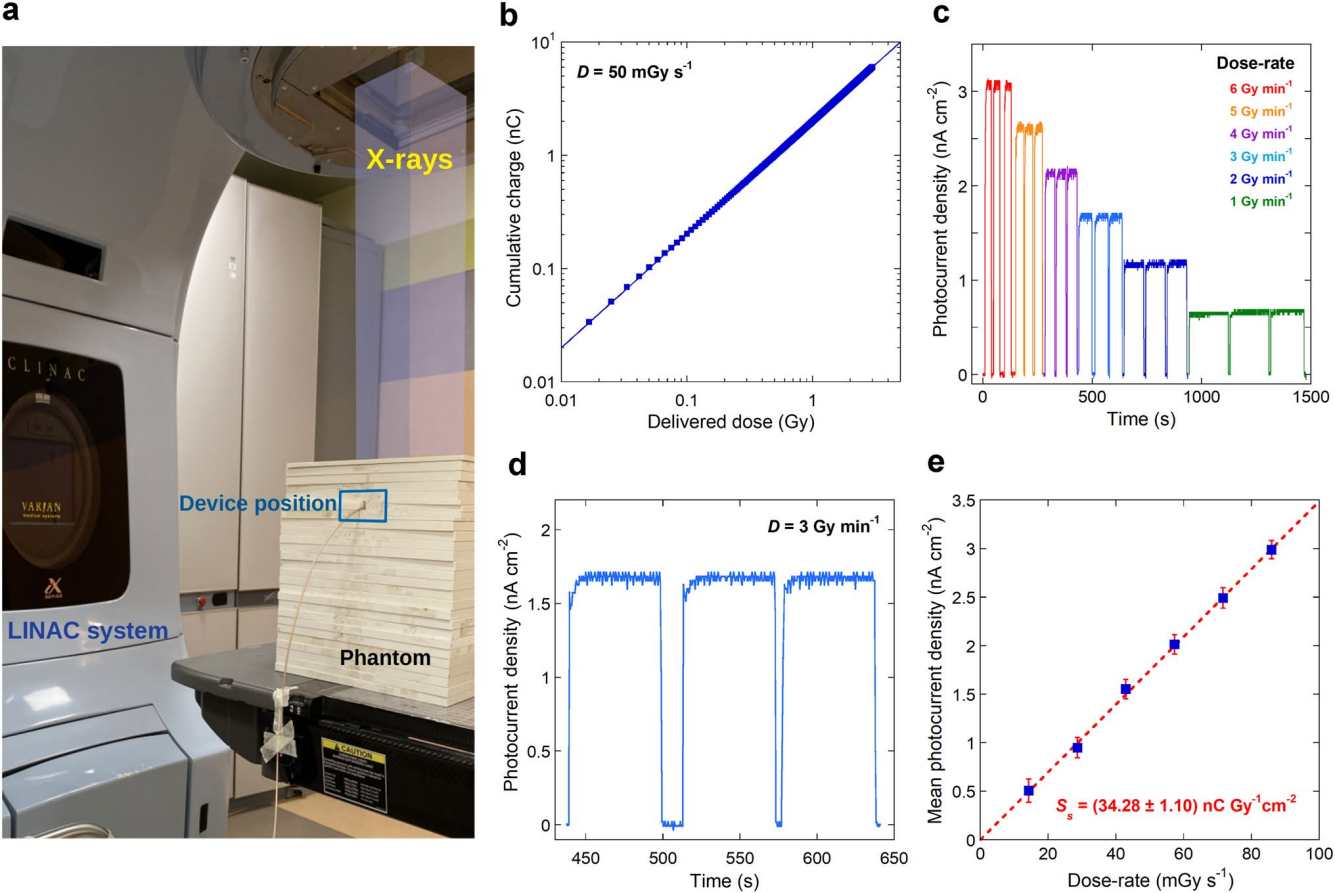
Validation of the X-ray detector in relevant environment. **a** Picture of the MeV-range X-ray irradiation setup. The solid phantom, consisting of 1 cm-thick PMMA (poly(methyl methacrylate) slabs, is used to ensure electronic equilibrium. **b** Cumulative charge collected as a function of the delivered dose up to 3 Gy. Error bars are smaller than the symbols. Blue continuous line indicates the best linear fit to the experimental data. **c** X-ray photocurrent density as a function of time recorded at different radiation dose rates. The “X-ray on” periods are programmed to deliver the same dose, whereas “X-ray off” periods vary between 5 and 20 s. **d** Detail of the measurement performed at the radiotherapy standard dose rate of 3 Gy min^−1^. **e** Mean values of the X-ray photocurrent density as a function of dose rate. Red dashed line indicates the best linear fit to the experimental data. The surface specific sensitivity is obtained from the slope of the red dashed fitting line

To further demonstrate the potentiality of the submicrometer-thick FAPbBr_3_ film as an active layer for X-ray detection, a device selected from the same batch described in Sect. 3.2 was validated in relevant environment with a medical linear accelerator (LINAC) routinely used for cancer treatment by radiation therapy with high-energy X-ray photons (Fig. 5a). The LINAC system was set to generate pulsed X-rays with energy up to 6 MeV. As can be seen from Fig. 5b, showing the results of a characterization performed at a fixed dose rate of 50 mGy s^−1^, excellent linearity is found between the cumulative charge collected by the detector and the dose delivered to the patient, which is an essential requirement for radiotherapy dosimetry. Figure 5c shows the photocurrent density measured during several beam on/off periods at different dose rates, varying in the range 1–6 Gy min^−1^ (Fig. 5d shows the detail of signals recorded at 3 Gy min^−1^, which is the typical dose rate used during medical treatments). Remarkably, the detector shows a perfectly reproducible response, which settles to a stable value after a few seconds. This quite a long settlement time, if compared to the case of keV-range X-rays (see Fig. S15) is however expected, because the LINAC system typically requires a few seconds to reach a stationary emission regime [61, 62], so we can infer that our detector is effective in real-time monitoring the delivered dose rate even during the LINAC transients.

The response linearity of the device under high-energy X-rays is further confirmed as a function of the dose rate, as can be seen from Fig. 5e, reporting the mean values of the photocurrent density extracted at each dose rate from data of Fig. 5c. As expected, the surface specific sensitivity is *S*_*s*_ = (34.28 ± 1.10) nC Gy^−1^ cm^−2^, which is significantly lower than that measured under continuous keV-range X-rays, due to the lower attenuation coefficient of the active layer under MeV-range radiations. However, it aligns consistently with the maximum theoretical value (*S*_*max*_ = 33 nC Gy^−1^ cm^−2^) estimated by considering the X-ray emission energy spectrum of the LINAC system (see Supplementary Note S3), highlighting once again the excellent charge collection efficiency of the device. More significantly, it is worth noting here that, being *S*_*s*_ ≈ *S*_*max*_, no photoconductive gain is present; this can be however explained by the high absorbed photon flux (about 10^11^ cm^−2^ s^−1^) under LINAC irradiation, which is well above the value (*φ* = 4.25 × 10^6^ cm^−2^ s^−1^) estimated to obtain *G* = *1* (Fig. S19). Therefore, it is reasonable to suppose that trap filling is rapidly reached, quenching the trap-assisted photoconductive gain. It is worth clarifying here that the higher absorbed photon flux under MeV-range X-rays is merely due to an experimental dose rate significantly higher than the case of keV-range X-rays, which overcompensates for the lower absorption capabilities.

## Conclusions

In summary, we developed a prototypal X-ray direct detector based on an optimized multilayer stack structure, exploiting a FAPbBr_3_ submicrometer-thick film as an active layer deposited on a mesoporous TiO_2_ scaffold. The device demonstrated self-powered operation, fast response speed, excellent linearity with the dose rate over more than three decades, an unprecedented bulk specific sensitivity of 7.28 C Gy^−1^ cm^−3^, and a limit of detection of 133 nGy s^−1^. What is most impressive, and unique in the world of perovskite-based X-ray detectors, is, however, the outstanding operational stability and reproducibility of the photocurrent signals measured in air (*i.e*., without encapsulation) over almost 4 weeks of uninterrupted irradiation. No signal drifts were indeed recorded within 24 h, and the average signal loss was evaluated to be only -1.3% after 26 days. In addition, the device did not show any significant structural damage after being exposed to a total ionizing dose of 192 Gy, retaining 97.2% of its external quantum efficiency and 94.2% of its specific detectivity, thus demonstrating excellent radiation hardness. Finally, the potentiality of the FAPbBr_3_ submicrometer-thick film as an active layer for superior-performance X-ray detectors was further assessed in relevant environment under MeV-range photons produced by a medical linear accelerator, showing how the proposed device stack could be reliably used as a dosimeter for radiation therapy.

These results point out that one of the most limiting “barrier to entry”, preventing MHPs from competing with other well-established materials for X-ray detectors, namely their long-term operational stability, has been overcome. Indeed, we demonstrate that the excellent detection properties can be not only retained after long-term storage, but also when operating uninterruptedly for several weeks, thus meeting all the conditions for a reliable device, and making MHP-based detectors moving forward on the path of market entry.

## Supplementary Information

Below is the link to the electronic supplementary material.

Supplementary file1 (DOCX 3078 kb)
