# Supplementary material for: Metal-Halide Perovskite Submicrometer-Thick Films for Ultra-Stable Self-Powered Direct X-Ray Detectors
Source: Nanomicro Lett. 2024 Apr 26;16:182. doi: 10.1007/s40820-024-01393-6 (PMC11052987; doi:10.1007/s40820-024-01393-6)
Supplement: Supplementary file 1 — Supplementary file1 (DOCX 3078 kb) [file 40820_2024_1393_MOESM1_ESM.docx]

Supplementary Information

**Metal-halide perovskite submicrometer-thick films for ultra-stable self-powered direct X-ray detectors**

Marco Girolami^1,^ *, Fabio Matteocci^2^, Sara Pettinato^1, 3^, Valerio Serpente^1^, Eleonora Bolli^1^, Barbara Paci^4^, Amanda Generosi^4^, Stefano Salvatori^1,3^, Aldo Di Carlo^2,4^, and Daniele M. Trucchi^1^

^1^CNR-ISM, Consiglio Nazionale delle Ricerche, Istituto di Struttura della Materia, Sede Secondaria di Montelibretti, DiaTHEMA Lab, Strada Provinciale 35D, 9, 00010 Montelibretti, Roma, Italy

^2^CHOSE – Centre for Hybrid and Organic Solar Energy, Department of Electronic Engineering, University of Rome ‘‘Tor Vergata’’, Via del Politecnico 1, 00133 Roma, Italy

Italy

^3^Faculty of Engineering, Università degli Studi Niccolò Cusano, Via don Carlo Gnocchi 3, 00166 Roma, Italy

^4^CNR-ISM, Consiglio Nazionale delle Ricerche, Istituto di Struttura della Materia, Area della Ricerca di Tor Vergata, SpecXLab, Via del Fosso del Cavaliere 100, 00133 Roma, Italy

*Corresponding author. E-mail: marco.girolami@ism.cnr.it


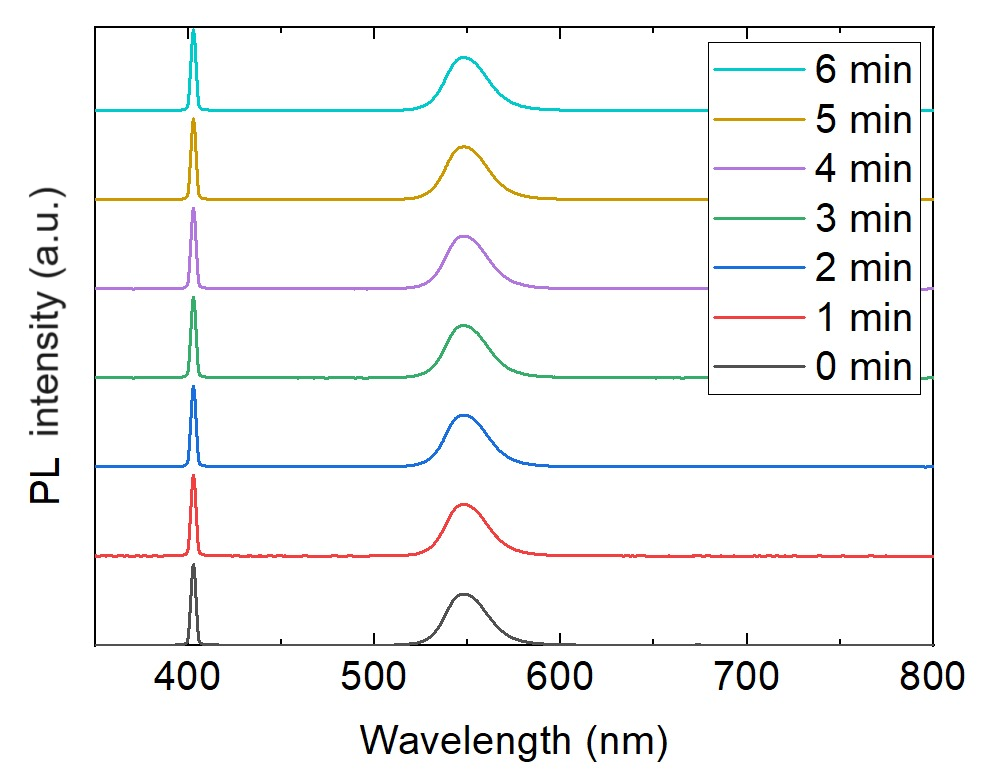


**Fig. S1.** Photoluminescence stability over time of the FAPbBr_3_ thin film. The spectra have been offset for clarity.


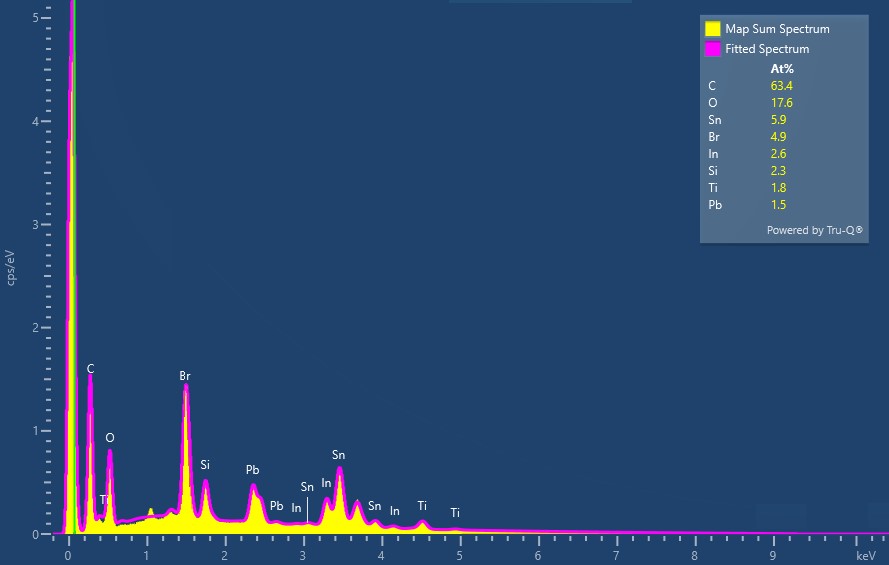


**Fig. S2.** EDX spectrum recorded from the complete device stack (Glass/FTO/c-TiO_2_/m-TiO_2_/FAPbBr_3_/PTAA/ITO) in the 0 – 10 keV range.


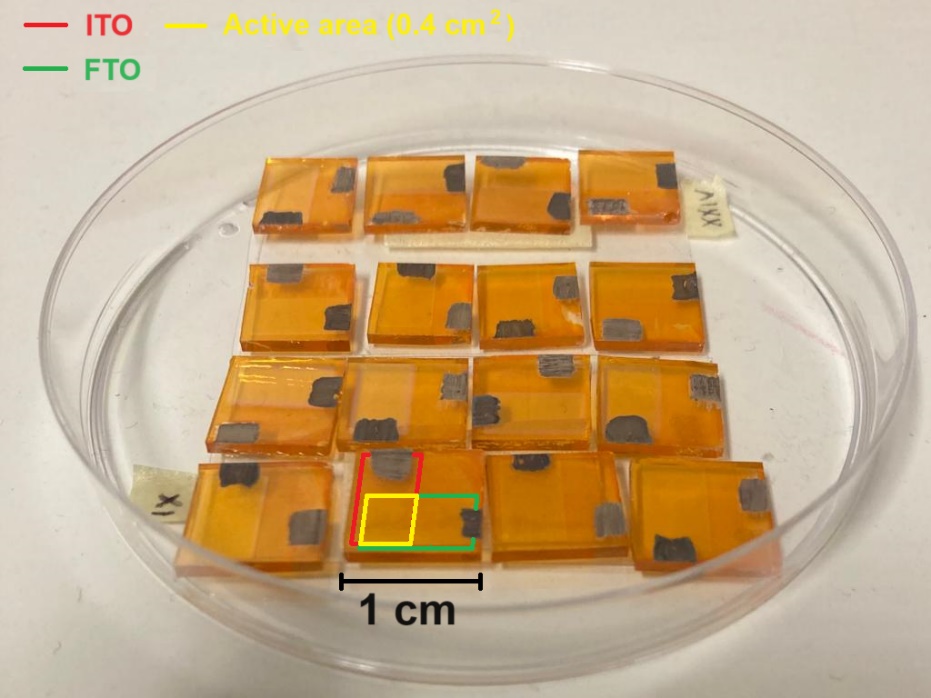


**Fig. S3.** Picture of 16 samples of the multi-layer stack used for the fabrication of the prototypal X-ray detector. Red and green boxes are a guide to the eye to indicate the lateral sizes of the collecting electrodes (red = ITO, green = FTO). Yellow box, obtained from the overlap of red and green boxes, visualizes the active area of the detector.


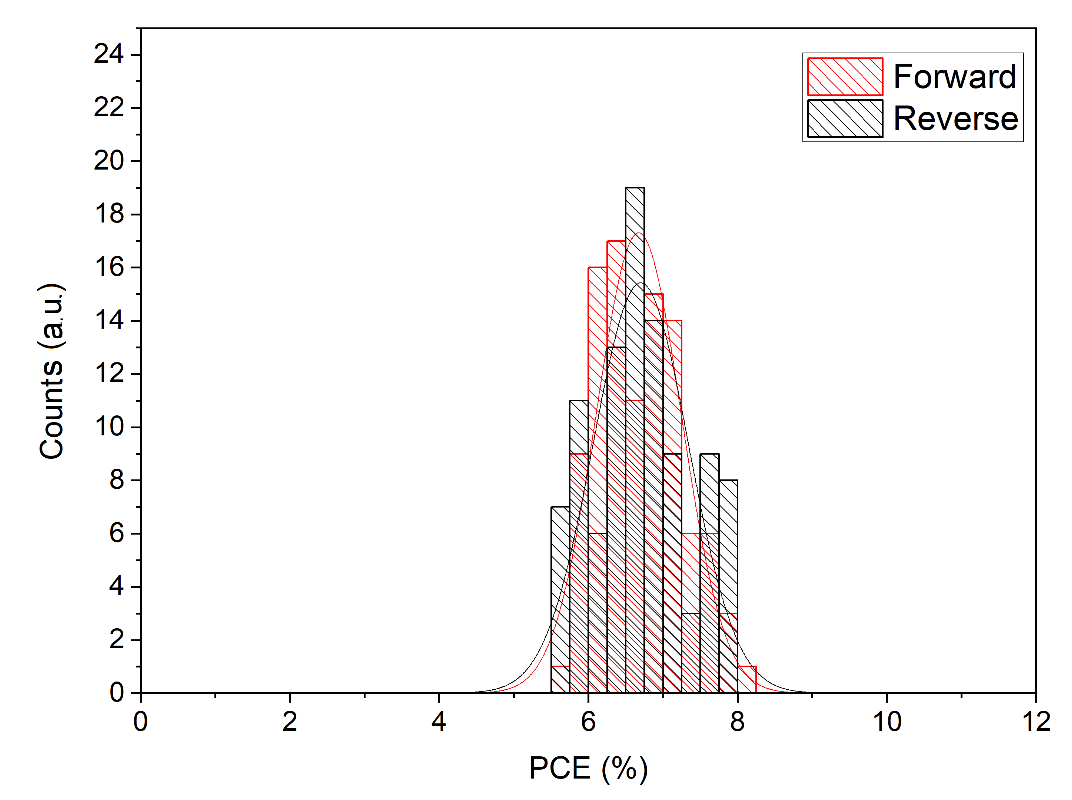


**Fig. S4.** Statistical dispersion of the power conversion efficiency (*PCE*) values obtained from a batch of 123 multi-layer stacks tested as semi-transparent perovskite solar cells (ST-PSCs), measured under forward (red bins) and reverse (black bins) scan directions at AM1.5G 1Sun illumination condition. The normal dispersion curves of *PCE* results are then calculated in order to extrapolate the average *PCE* values for each scan direction.

**
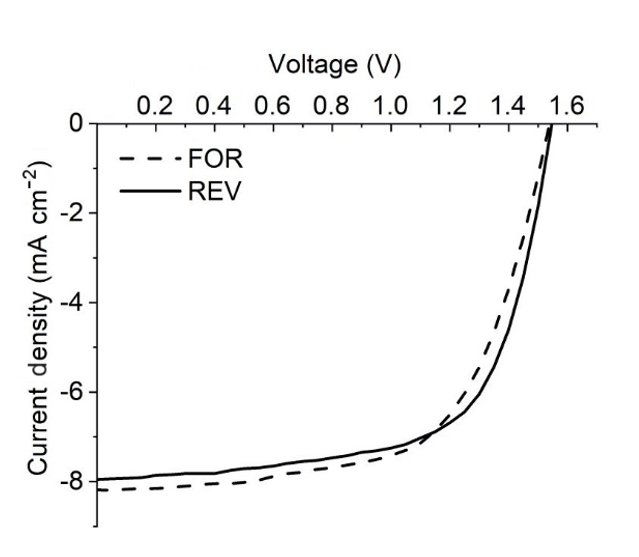
**

**Fig. S5*.*** *J-V* characteristics of the best performing ST-PSC measured under forward (dashed line) and reverse (solid line) scan directions at AM1.5G 1 Sun illumination condition.

**
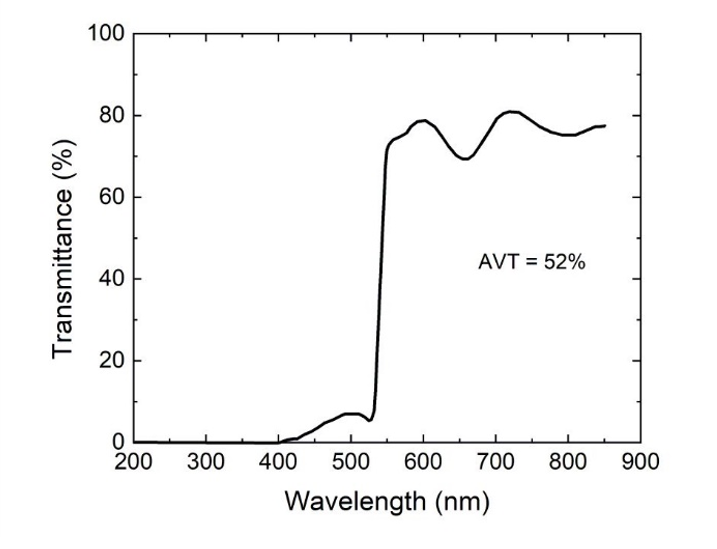
**

**Fig. S6.** Transmittance of the full ST-PSC device in the 200 – 850 nm wavelength range. The average visible transmittance (*AVT*) is 52%.


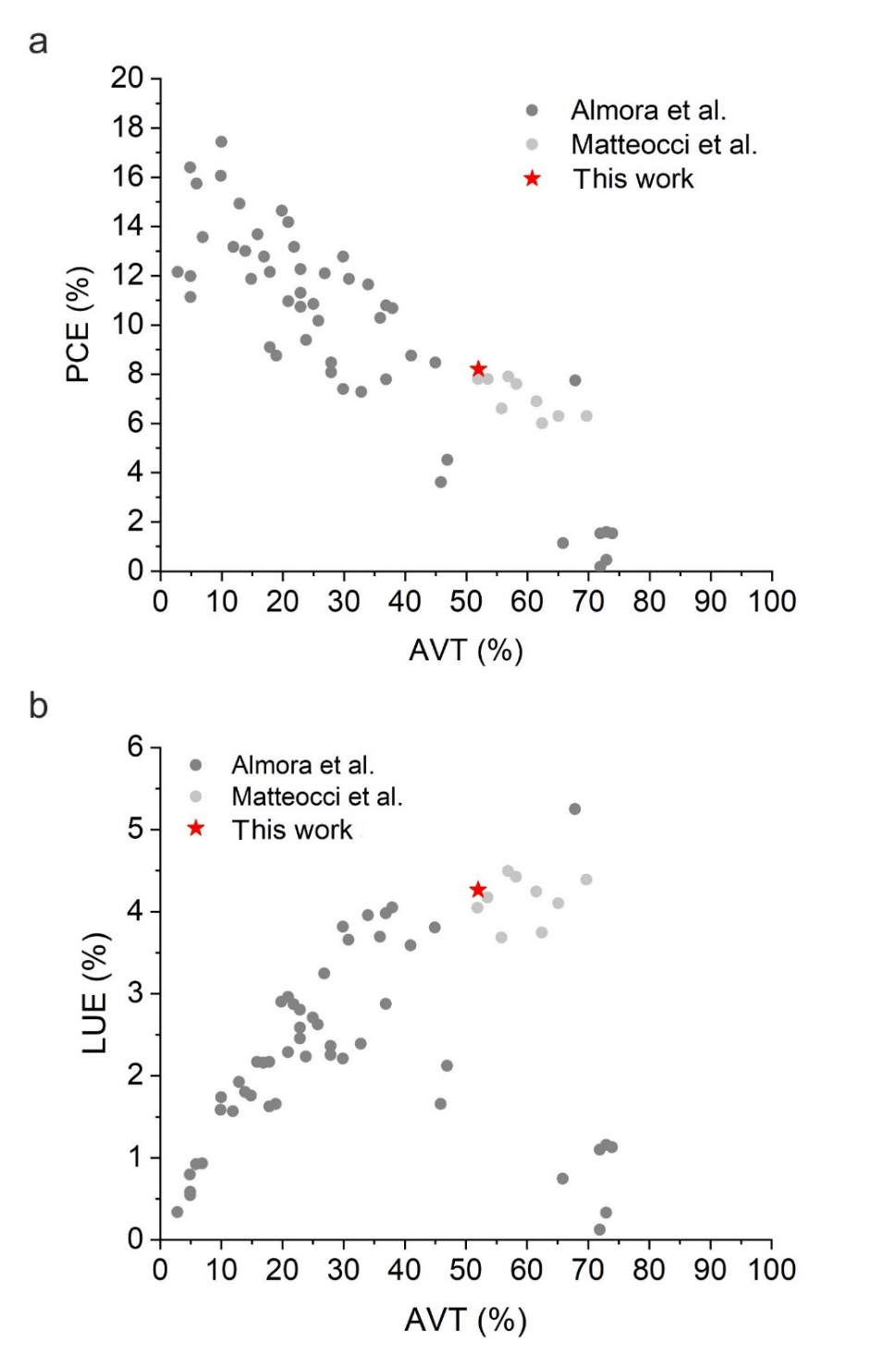


**Fig. S7.** a) *PCE* vs. *AVT* and b) *LUE* (Light Utilization Efficiency) vs. *AVT* graphs showing the state-of-the-art results for ST-PSCs reported by Almora et al. (dark gray dots) [S1] and Matteocci et al. (light gray dots) [S2] on MAPbBr_3-x_Cl_x_, and the results presented in this work (red stars).

^^

**Fig. S8.** Amplitude of the modulated photocurrent of two different FAPbBr_3_ thin film devices (FS-VI and FS-VIII) in the 200 – 700 nm wavelength range at *V*_B_ = 0 V. Peaks appearing in the 300 – 500 nm are caused by the strong spectral lines of the Xe-Hg lamp, whereas the peak at about 550 nm is the excitonic peak.

**Fig. S9.** Phase of the modulated photocurrent of two different FAPbBr_3_ thin film devices (FS-VI and FS-VIII) in the 200 – 700 nm wavelength range at *V*_B_ = 0 V.

**Fig. S10.** Responsivity of two different FAPbBr_3_ thin film devices (FS-VI and FS-VIII) in the 400 – 700 nm wavelength range at *V*_B_ = 0 V.

^^

**Fig. S11.** Specific detectivity of two different FAPbBr_3_ thin film devices (FS-VI and FS-VIII) in the 400 – 700 nm wavelength range at *V*_B_ = 0 V.

**Fig. S12.** Dark current density (*J_d_*) vs. bias voltage (*V*_B_) measured for two different FAPbBr_3_ thin film devices (FS-VI and FS-VIII) in forward bias mode (up to +1.5 V) and in reverse bias mode (down to -1.5 V).

Note: The semilogarithmic *J_d_* vs. *V*_B_ plots show two distinguishable bias-dependent regions in forward mode. The low bias region (*V*_B_ < 1 V) is dominated by a high shunt resistance, reflecting a very low leakage current between the electric contacts and the active layer. In the high bias region (*V*_B_ > 1 V), plots are almost parallel and follow an exponential behaviour due to the diode characteristics. Conversely, in reverse mode, only the shunt resistance regime is present, implying that the bias voltage is not intense enough to trigger breakdown. In addition, absolute bias voltage being equal, the dark current is always lower in reverse mode than in forward mode.

^^

**Fig. S13.** Amplitude of the modulated photocurrent of a FAPbBr_3_ thin film device (FS-VI) in the 300 – 800 nm wavelength range measured at two different bias voltages: 0 V (blue curve) and -0.5 V (red curve). Peaks appearing in the 300 – 500 nm wavelength range are caused by the strong spectral lines of the Xe-Hg lamp, whereas the peak at about 550 nm is the excitonic peak.

^
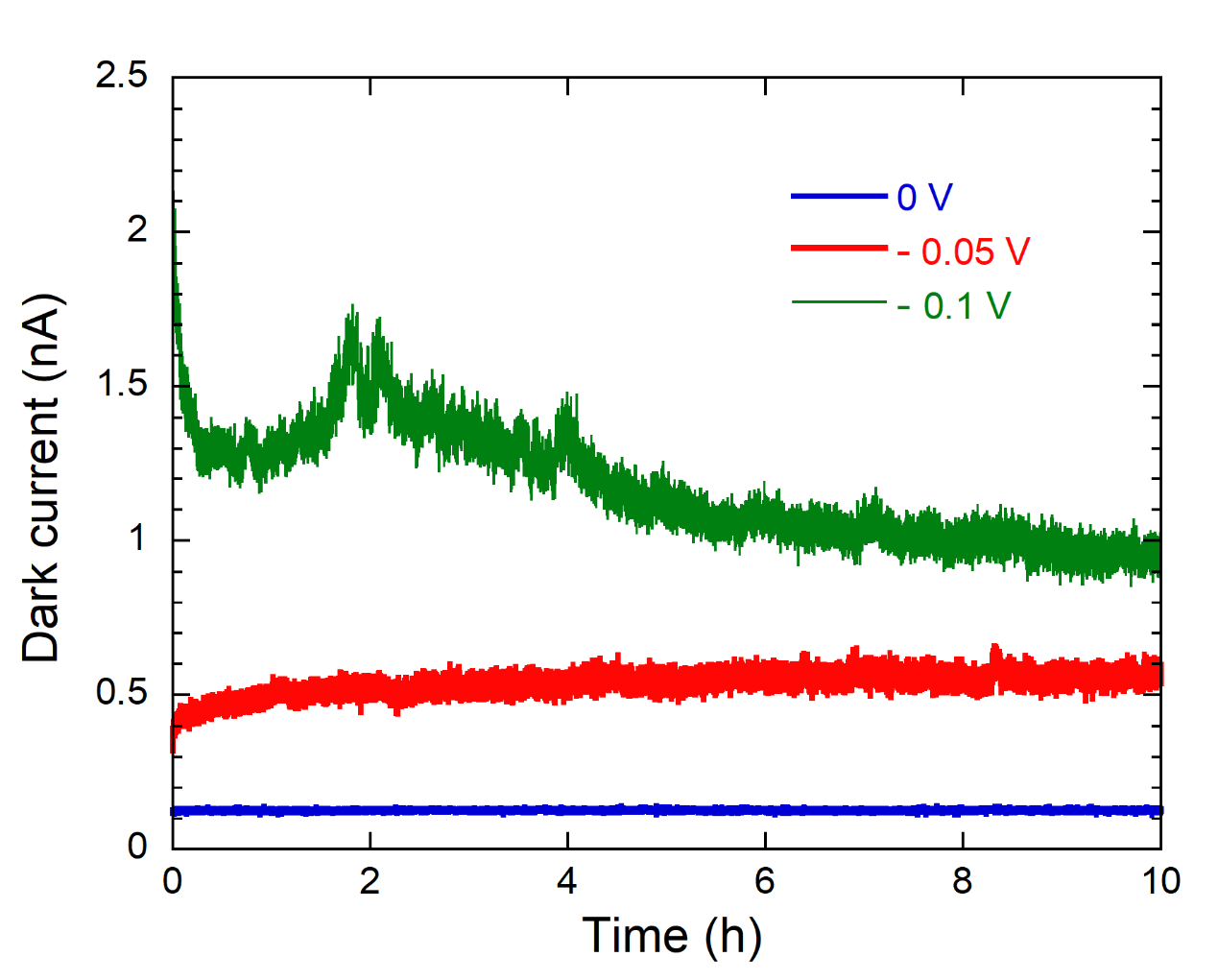
^

**Fig. S14.** Dark current measured as a function of time for a FAPbBr_3_ thin film device (FS-VI) at three different bias voltages: 0 V (blue curve), -0.05 V (red curve9, and -0.1 V (green curve).

.

^^

**Fig. S15.** Response time of a FAPbBr_3_ thin film device (FS-VI) to X-rays at *V*_B_ = 0 V. Acceleration voltage was set to 40 kV, tube current was set to 15 mA, corresponding to a dose-rate of 69.3 μGy s^-1^. The delay time between two measurement points was set to 0.25 s.

Note: The photocurrent is generated when X-rays are switched on, then the signal drops to zero when X-rays are switched off. Therefore, both the rise (*t_r_*) and decay (*t_d_*) time include the response time of the detector and the on/off time of the X-ray tube. The signal rises from 10% to 90% of its stationary value within the delay time between three measurement points, so the upper limit for *t_r_* is 0.5 s, whereas it decays from 90% to 10% of its stationary value within the delay time between two measurement points, so the upper limit for *t_d_* is only 0.25 s.

**Fig. S16.** Total current (dark current + photocurrent) measured at *V*_B_ = 0 V for a FAPbBr_3_ thin film device (FS-VI) when irradiated at the lowest dose-rate (644 nGy s^-1^) available with the adopted X-ray source. The shutter is opened at *t* = 100 s, and the current rises immediately from its value in dark conditions (about 127 pA) to a stable value of about 175 pA.

**Supplementary Note 1: Estimation of the upper sensitivity limit under keV-range X-rays**

The upper theoretical limit of the specific surface sensitivity *S_max_* (C Gy^-1^ cm^-2^) of a detector for ionizing radiation can be derived from the maximum theoretical photocurrent density *J_max_* (A cm^-2^) obtainable at a given dose-rate in air *D* (J kg^-1^) :

$$S_{max}=\frac{J_{max}}{D}$$

The maximum theoretical photocurrent is given by:

$$J_{max}=e\beta=e\frac{E_{ph}}{E_{i}}$$

where *e* = 1.61 × 10^-19^ C is the electron charge, *ϕ* (cm^-2^ s^-1^) is the flux of photons effectively absorbed by the active layer per unit time (*i.e.*, the absorbed photon flux), and *β* = *E_ph_/E_i_* is the maximum number of electron-hole pairs photogenerated by a single photon with energy *E_ph_*. The quantity *E_i_* is the electron-hole pair creation energy, which can be expressed (in eV) as *E_i_* = 2*E_g_* + 1.43 (where *E_g_* is the bandgap energy), according to an empirical model [S3] used to successfully predict *E_i_* for most of the semiconductors, and therefore widely employed in the field of X-ray detectors.

Aimed at calculating the absorbed photon flux *ϕ*, it is necessary to evaluate the dose-rate effectively absorbed by the active layer (*D*_P_), which can be obtained by the dose-rate measured in air (*D*) by the simple proportionality relationship *D*_P_/*D* = *μ*_P_/*μ*, where *μ*_P_ and *μ* (cm^2^ g^-1^) are the mass attenuation coefficients of the active layer material and dry air, respectively, at the photon energy *E_ph_*. In this way, by supposing that X-rays are uniformly absorbed within the submicrometer-thick active layer, the absorbed photon flux can be expressed as:

$$=\frac{D_{P}\rho d}{E_{ph}}=\frac{D\frac{\mu_{P}}{\mu}\rho d}{E_{ph}}$$

where *ρ* (g cm^-3^) and *d* (cm) are the active layer density and thickness, respectively.

By substitution, we can now obtain two simple operative expressions to evaluate *J_max_* and *S_max_* by only measuring the dose-rate in air *D*:

$$J_{max}=\frac{eD\mu_{P}\rho d}{\mu E_{i}}$$

$$S_{max}=\frac{e\mu_{P}\rho d}{\mu E_{i}}$$

In the case of the device introduced in this work, the active material is a FAPbBr_3_ thin film with thickness *d* = 255 × 10^-7^ cm. For the estimation of *J_max_*, we assumed [S4] *ρ* = 3.79 g cm^-3^, whereas *μ*_P_ was obtained by using the XCOM software [S5] provided by NIST (National Institute of Standards and Technology). Figure S17 reports the mass attenuation coefficient and the relative X-ray absorption (*i.e.*, the fraction of incident photons effectively absorbed) of FAPbBr_3_ in the 1 keV – 10 MeV energy range.

**Fig. S17.** Relative X-ray absorption (blue curve) and mass attenuation coefficient (red curve) of FAPbBr_3_ in the 1 keV – 10 MeV energy range. The relative X-ray absorption coefficient (𝜖) was directly obtained from *μ*_P_ by applying the relationship: 𝜖 = 1 – exp(–*μ*_P_ *ρd)*.

By considering a photon energy *E_ph_* = 8.05 keV, which is the most probable energy in the case of a Cu-target X-ray tube, the mass attenuation coefficient is *μ*_P_ = 140.80 cm^2^ g^-1^, corresponding to a relative X-ray absorption 𝜖 = 1.36% for a 255 nm-thick film. At the same photon energy (8.05 keV), the mass attenuation coefficient of dry air [S6] is *μ* = 9.92 cm^2^ g^-1^. Finally, being the bandgap energy *E_g_* = 2.28 eV, the estimated electron-hole pair creation energy is *E_i_* = 2 ⋅ 2.28 + 1.43 = 5.99 eV = 9.58 × 10^-19^ J.

By substituting all the numerical values into the *S_max_* equation we obtain:

$$S_{max}=\frac{e\mu_{P}\rho d}{\mu E_{i}}=0.23 \mu C {Gy}^{-1}{cm}^{-2}$$

^^

**Fig. S18.** Semilogarithmic plot of the photoconductive gain factor (*G*) as a function of the absorbed photon flux (*ϕ*) for *ϕ* > 10^4^ cm^-2^ s^-1^. Red dashed line indicates the best fit to data obtained by using a mono-exponential decay equation.


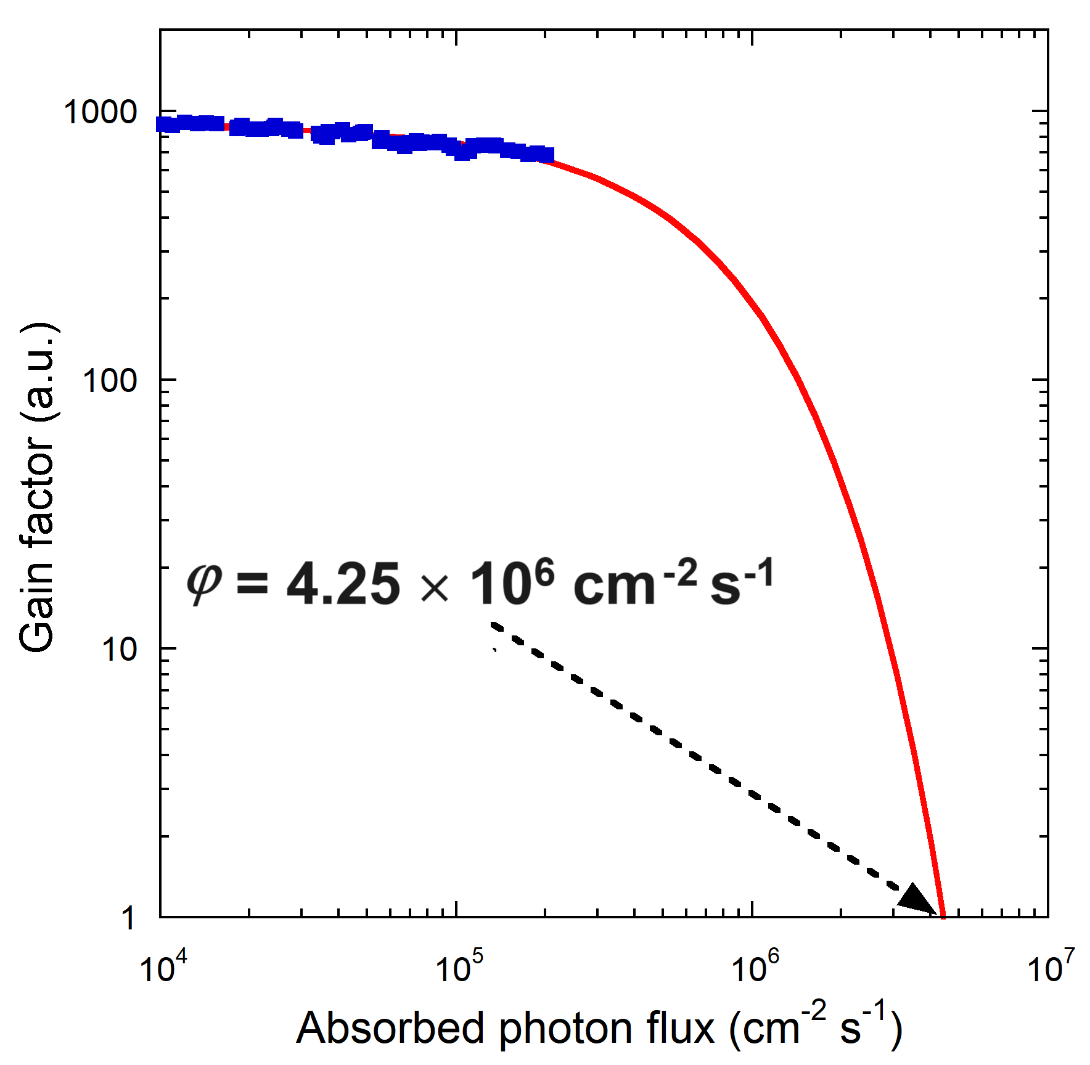


**Fig. S19.** Extrapolation of gain factor data for high absorbed photon flux values. Solid red line indicates the best exponential decay fit to data. Dashed black arrow indicates the extrapolated absorbed photon flux corresponding to *G* = 1.

**Fig. S20.** Relative X-ray absorption (blue plot) and maximum theoretical sensitivity (red plot) at a photon energy *E_ph_* = 8.05 keV of a FAPbBr_3_ thin film as a function of thickness in the 250 – 1000 nm range.

^^

**Fig. S21.** Responsivity of a FAPbBr_3_ thin film device (FS-VI) in the UV-Vis-NIR wavelength range (250 – 800 nm) at *V*_B_ = 0 V. Blue curve refers to measurements performed before starting X-ray characterization, whereas red curve has been measured after 26 days of uninterrupted X-ray irradiation.

**Fig. S22.** Specific detectivity of a FAPbBr_3_ thin film device (FS-VI) in the UV-Vis-NIR wavelength range (250 – 800 nm) at *V*_B_ = 0 V. Blue curve refers to measurements performed before starting X-ray characterization, whereas red curve has been measured after 26 days of uninterrupted X-ray irradiation.

^^

**Fig. S23.** Logarithmic plot of the total current (dark current + photocurrent) measured at *V*_B_ = 0 V in the final 200 s of the 26-day long X-ray irradiation period, showing a slight increase of the average dark current with respect to the initial value (Fig. 4d).

**Fig. S24.** Dark current measured as function of time for two FAPbBr_3_ thin film-based device stacks: with (black plot) and without (red plot) the mesoporous TiO_2_ scaffold.

**Fig. S25.** Total current (dark current + photocurrent) measured under X-ray irradiation at different dose-rates for two FAPbBr_3_ thin film-based device stacks: with (black plot) and without (red plot) the mesoporous TiO_2_ scaffold. X-ray tube voltage was set to 40 kV. Dose-rate was varied every 100 s in the 3.3 – 31.3 μGy s^-1^ range, with dose-rate steps of 4 μGy s^-1^. Dashed lines are a visual guide to indicate the dark current drift: no drift for the device with m-TiO_2_, and a positive drift (red arrow) for the device without m-TiO_2_. It is worth observing that the minimum dose-rate (3.3 μGy s^-1^) is detected by the device without m-TiO_2_ only in the rising phase of the curve; conversely, in the falling phase, the photocurrent signal is completely masked by the background noise (blue circle), due to the positive drift of the dark current.

**Supplementary Note 2: Estimation of the electron trap density**

The electron trap density of the FAPbBr_3_ 255-nm thick active layer of the X-ray detector was estimated by performing space-charge-limited-current (SCLC) measurements on the electron-only device. The current flowing in the device was measured in dark conditions as a function of the applied bias voltage in the 0 – 3 V range (corresponding to an applied electric field approximately in the 0 – 11.8 V μm^-1^ range) by means of a Keithley 6517A electrometer. Results are shown in Fig. S26.


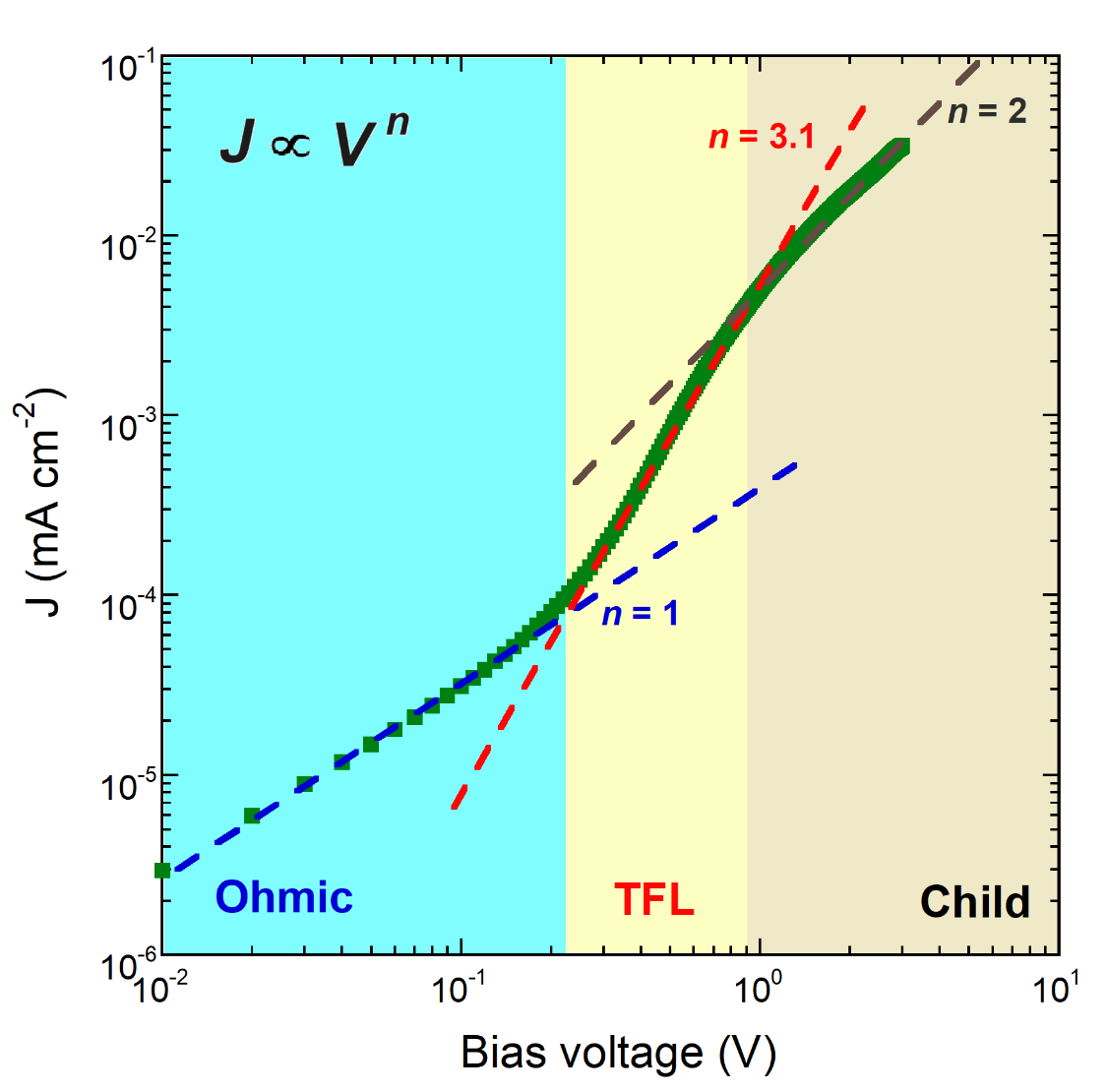


**Fig. S26**. Log-log plot of the dark current density of a FAPbBr_3_ thin film-based electron-only device as a function of the applied bias voltage. The three coloured boxes are a visual guide to define the voltage ranges of the three identified charge transport regimes: Ohmic (light blue), trap-filled-limited (light yellow), and Child (gray). Dashed lines indicate the best power fits (*J* ∝ *V^n^*) to the experimental data. The values of *n* extracted from the fitting process are also shown.

As can be seen, three different charge transport regimes can be identified. For *V* < 0.22 V the dark current density increases linearly with the applied bias voltage (*J* ∝ *V*) according to Ohm’s law. At *V =* 0.22 V, a steep increase in dark current is observed due to trap filling (*J* ∝ *V^n^*, with *n* = 3.1). Then, when all the traps are filled (*V* > 0.9 V), space-charge-limited-current regime (also known as Child’s regime) is entered, and the dark current increases quadratically with the applied bias voltage (*J* ∝ *V*^2^).

The electron trap density *n_t_* can be calculated using the following equation [S7]:

$$n_{t}=\frac{2\varepsilon_{0}\varepsilon_{r}V_{TFL}}{ed^{2}}$$

where *V_TFL_* = 0.22 V is the trap-filled limit voltage (denoting the critical point from Ohmic to trap-filled-limited regime), *ε*_0_ = 8.85 × 10^-14^ C V^-1^ cm^-1^ is the vacuum permittivity, *ε_r_* = 4.12 is the relative permittivity of FAPbBr_3_ [S8], *e* = 1.61 × 10^-19^ C is the electron charge and *d* = 255 ± 5 nm is the film thickness. By substituting the numerical values, we obtain: *n_t_* = (1.54 ± 0.06) × 10^15^ cm^-3^.

**Supplementary Note 3: Assessment of the detector sensitivity to pulsed MeV-range X-rays**

A medical linear accelerator (LINAC) emits X-rays with a broad continuous energy spectrum (*Bremsstrahlung* radiation). The maximum energy (*E_max_*) corresponds to the accelerating voltage of the electrons striking a heavy metal target (W in our case). The total X-ray photon flux incident on the detector can be calculated as follows:

$$\varphi_{in}=\int_{0}^{E_{max}} \frac{\partial\varphi_{in}\left( E_{ph} \right)}{\partial E_{ph}}{dE}_{ph}$$

where *E_ph_* is the photon energy, and *∂φ_in_(E_ph_)*/*∂E_ph_* is the incident photon flux per unit energy expressed as:

$$\frac{\partial\varphi_{in}\left( E_{ph} \right)}{\partial E_{ph}}=\frac{DS(E_{ph})}{\mu(E_{ph})E_{ph}}$$

where *D* is the dose-rate, *µ(E_ph_)* is the mass attenuation coefficient of dry air at a given X-ray energy *E_ph_* , and *S(E_ph_)* represents the *Bremsstrahlung* spectrum distribution with:

$$\int_{0}^{E_{max}} S\left( E_{ph} \right){dE}_{ph}=1$$

The spectral distribution of the *Bremsstrahlung* radiation emitted by a W target under 6 MeV electrons, as simulated by GEANT4 code [S9], is reported in Fig. S27.

The fraction of photons effectively absorbed by the FAPbBr_3_ active layer is given by:

$$\epsilon(E_{ph})=1-e^{-\mu_{P}\left( E_{ph} \right)\rho d}$$

where *µ_P_(E_ph_)*, *ρ*, and *d* are the X-ray mass attenuation coefficient at a given X-ray energy *E_ph_*, density, and thickness of the FAPbBr_3_ active layer, respectively. In our case, *ρ* = 3.79 g cm^-3^ and *d* = 255 × 10^-7^ cm. Figure S28 shows the calculated X-ray attenuation coefficients for FAPbBr_3_ (red curve) and dry air (blue curve) in the 1 keV – 10 MeV energy range.

**Fig. S27.** GEANT4 simulation of the spectrum of X-ray *Bremsstrahlung* radiation emitted by a W target under 6 MeV electrons.

**Fig. S28**. Mass attenuation coefficients as a function of the X-ray photon energy for FAPbBr_3_ (red curve) and dry air (blue curve). Calculations were performed by using the NIST database [S5, S6].

The absorbed photon flux per unit energy can be calculated as:

$$\frac{\partial\varphi\left( E_{ph} \right)}{\partial E_{ph}}=\frac{DS(E_{ph})}{\mu(E_{ph})E_{ph}}\left[ 1-e^{-\mu_{P}\left( E_{ph} \right)\rho d} \right]$$

Results for the FAPbBr_3_ active layer, with nominal dose-rates ranging between 1 Gy min^-1^ and 6 Gy min^-1^ are reported in Fig. S29:


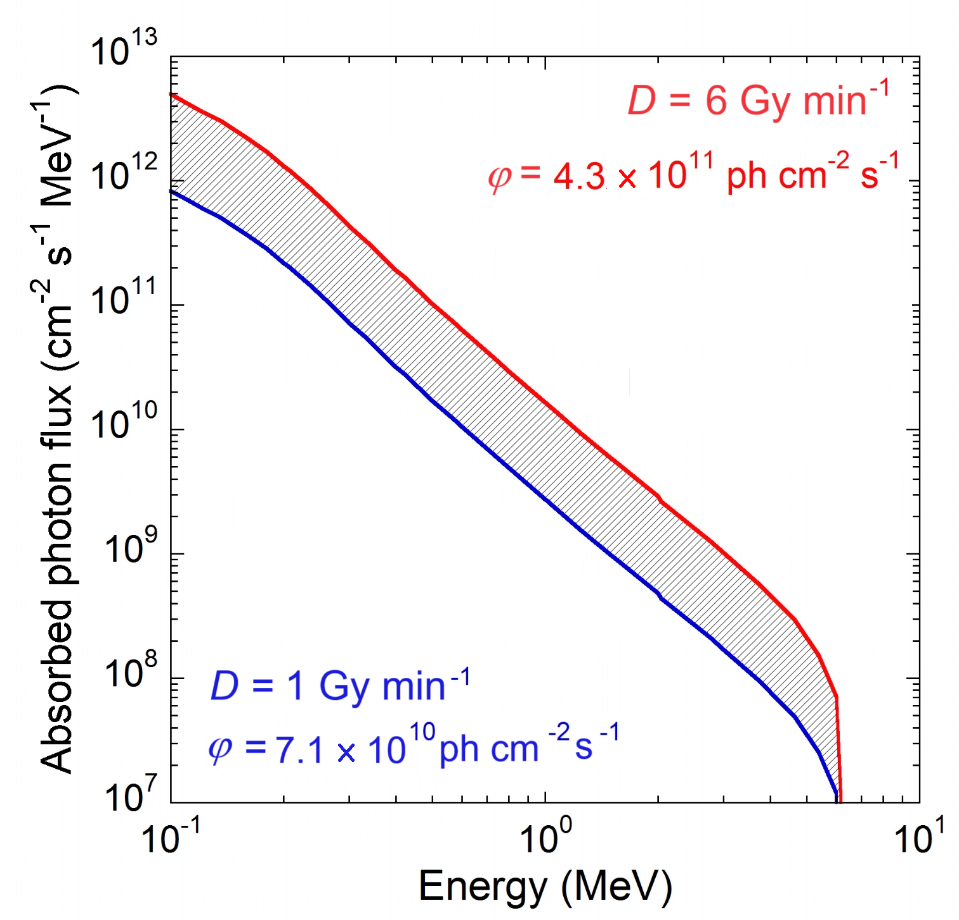


**Fig. S29**. Absorbed photon flux per unit energy as a function of X-ray energy for a 255 nm thick FAPbBr_3_ layer. Red and blue curves refer to the values estimated for the upper (6 Gy min^-1^) and the lower (1 Gy min^-1^) investigated dose-rates, respectively. The shaded area refers to values estimated for 1 < *D* < 6 Gy min^-1^.

The absorbed photon flux can be finally derived from the following equation.

$$\varphi=\int_{0}^{E_{max}} \frac{DS(E_{ph})}{\mu(E_{ph})E_{ph}}\left[ 1-e^{-\mu_{P}\left( E_{ph} \right)\rho d} \right]dE_{ph}$$

By substituting *E_max_* = 6 MeV , we obtain values ranging between 7.1 × 10^10^ cm^-2^ s^-1^ and 4.3 × 10^11^ cm^-2^ s^-1^ in the 1 – 6 Gy min^-1^ dose-rate range.

The previous equation also allows us to estimate the photocurrent density *J_ph_*, expressed as the rate of electron-hole pairs generated by the absorbed photons and collected by the electrodes:

$$J_{ph}=e\eta\int_{0}^{E_{max}} \frac{\partial\varphi\left( E_{ph} \right)}{\partial E_{ph}}\frac{E_{ph}}{E_{i}}dE_{ph}$$

where *e* = 1.6 × 10^-19^ C is the electron charge, *η* is the charge collection efficiency, and *E_i_* = 5.99 eV is the electron-hole pair creation energy for FAPbBr_3_. The maximum theoretical photocurrent density (*J_max_*) at the different dose-rates, and consequently the maximum theoretical specific surface sensitivity (*S_max_* = *J_max_* /*D*), can be obtained by assuming *η* = 1. For instance, when the dose rate is *D* = 6 Gy min^-1^, we obtain *J_max_* = 2.84 nA cm^-2^ and *S_max_* = 33.02 nC Gy^-1^ cm^-2^, which are remarkably consistent with the values obtained experimentally (*J_ph_* = 3.11 ± 0.10 nA cm^-2^ and *S*_s_ = 34.28 ± 1.10 nC Gy^-1^ cm^-2^, respectively).

**References**

[S1] O. Almora, D. Baran, G. C. Bazan, C. I. Cabrera, S. Erten-Ela, K. Forberich, F. Guo, J. Hauch, A. W. Y. Ho-Baillie, T. J. Jacobsson, R. A. J. Janssen, T. Kirchartz, N. Kopidakis, M. A. Loi, R. R. Lunt, X. Mathew, M. D. McGehee, J. Min, D. B. Mitzi, M. K. Nazeeruddin, J. Nelson, A. F. Nogueira, U. W. Paetzold, B. P. Rand, U. Rau, H. J. Snaith, E. Unger, L.Vaillant-Roca, C.Yang, H.-L. Yip, C. J. Brabec, *Adv. Energy Mater.* **2023**, *13*, 2203313. <https://doi.org/10.1002/aenm.202203313>

[S2] F. Matteocci, D. Rossi, L. A. Castriotta, D. Ory, S. Mejaouri, M. Auf der Maur, F. Sauvage, S. Cacovich, A. Di Carlo, *Nano Energy* **2022**, *101*, 107560. <https://doi.org/10.1016/j.nanoen.2022.107560>

[S3] R. Devanathan, L. R. Corrales, F. Gao, W. J. Weber, *Nucl. Instrum. Methods Phys. Res. A* **2006**_,_ *565*, 637–649. <https://doi.org/10.1016/j.nima.2006.05.085>

[S4] Y. Rakita, S. R. Cohen, N. K. Kedem, G. Hodes, D. Cahen, *MRS Commun*. **2015**, *5*, 623–629. <https://doi.org/10.1557/mrc.2015.69>.

[S5] NIST (National Institute of Standards and Technology) – XCOM database, <https://physics.nist.gov/PhysRefData/Xcom/html/xcom1.html>, accessed: October, **2023**.

[S6] J. H. Hubbell, S. M. Seltzer, X-Ray Mass Attenuation Coefficients, NIST Standard Reference Database 126 (NISTIR 5632), <https://dx.doi.org/10.18434/T4D01F>, accessed: October, **2023**.

[S7] R. H. Bube, *J. Appl. Phys.* **1962**, *33*, 1733–1737. <https://doi.org/10.1063/1.1728818>

[S8] R. Mayengbam, J. T. Mazumder, *Int. J. Energy Res.* **2022**, 46, 17556–17575. <https://doi.org/10.1002/er.8422>

[S9] I. I. Haysak, O. V. Takhtasiev, J. Khushvaktov, A. A. Solnyshkin, A. Tanchak, R. R. Holomb, K. Katovsky, in *Proceedings of the 2020 21^st^ International Scientific Conference on Electric Power Engineering (EPE)*, Prague, Czech Republic, 19–21 October **2020**, 1–4. <https://doi.org/10.1109/EPE51172.2020.9269252>.
